# A new fuzzy rule based multi-objective optimization method for cross-scale injection molding of protein electrophoresis microfluidic chips

**DOI:** 10.1038/s41598-022-15935-8

**Published:** 2022-08-01

**Authors:** Zhiying Shan, Wangqing Wu, Yihua Lei, Baishun Zhao

**Affiliations:** 1 State Key Laboratory of High Performance Complex Manufacturing, Central South University, Changsha, 410083 China; 2 School of Mechanical and Electrical Engineering, Central South University, Changsha, 410083 China

**Keywords:** Biomedical engineering, Mechanical engineering

## Abstract

Injection molding is one of the most promising technologies for the large-scale production and application of polymeric microfluidic chips. The multi-objective optimization of injection molding process for substrate and cover plate on protein electrophoresis microfluidic chip is performed to solve the problem that the forming precision is difficult to coordinate because of the cross-scale structure characteristics for chip in this paper. The innovation for this research is that an optimization approach and a detailed fuzzy rule determination method are proposed in multi-objective optimization for protein electrophoresis microfluidic chip. In more detail, firstly, according to the number and level of process parameters, the orthogonal experimental design is carried out. Then, the experiments are performed. Secondly, the grey relational analysis (GRA) approach is employed to process the response data to gain the grey relational coefficient (GRC). Thirdly, the grey fuzzy decision making method which combines triangular membership function and gaussian membership function is adopted to obtain the grey fuzzy grade (GFG). After that, the optimal scheme of process parameters was predicted by the grey fuzzy grade analysis. Finally, the superiority of Taguchi grey fuzzy decision making method are verified by comparing the results of original scheme, optimal scheme and prediction scheme. As a result, compared with the original design, the residual stress of substrate plate (RSS), residual stress of cover plate (RSC), warpage of substrate plate (WS), warpage of cover plate (WC) and replication fidelity of microchannel for substrate plate (RFM) on the prediction scheme for Taguchi grey fuzzy decision making method were reduced by 32.816%, 29.977%, 88.571%, 74.390% and 46.453%, respectively.

## Introduction

In recent years, with the progress of science and technology, the requirements of laboratory testing technology are becoming higher and higher (shorter detection time, Higher detection sensitivity, More automation, etc.), especially in chemical analysis, medical testing, life science and other fields^1,2^. Microfluidic chips have been attracted great attention worldwide and have high application prospects in the above fields due to the analysis and detection process is characterized by miniaturization, integration, rapidness, low cost, few reagents and high throughput^3^.

At present, most of the research on microfluidic chip is focused on the molding technology^4–9^, bonding technology^10,11^ and application technology^12,13^. The main molding methods of microfluidic chips include hot pressing, injection molding and laser ablation. As the most widely used technology, injection molding has the advantages of low cost, high efficiency, and high precision. Injection molding is a key technology for mass production and application of microfluidic chips, many scholars have carried out research on chip of cross-scale injection molding. Calaon et al. evaluated the key factors affecting the reproduction quality of microchannels and designed the microfluidic chip^14^. Xie et al. adopted single-factor experiment method to eliminate the bubble defects in the injection molding process of the chip, and obtained a microfluidic chip with good surface quality and high dimensional accuracy^15^. Jiang et al. optimized the microchannel cross section size of triple-channel microfluidic chip and obtained the optimal process parameters^16^. Marson et al. realized low warpage of microfluidic chip by optimizing the design of injection mold^17^. Jena et al. researched and optimized the residual stress of microfluidic chip^18^. Due to the narrow process window and many parameters, the problem of how to obtain precise chip forming process efficiently has not been solved yet. During the forming process, the cross-scale geometric deviation between the macroscopic warping deformation of chip and the deformation of microchannel is difficult to coordinate, and the accuracy is difficult to ensure. Meanwhile, a qualified microchannel chip needs not only high replication fidelity of microchannel, but also good bonding quality. However, the warpage and residual stress of substrate and cover plate for microfluidic ship will seriously affect the bonding quality of the chip, which will lead to non-sealing and deformation of the chip. Most of the existing researches only consider one objective to carry out single-objective optimization^16–18^. Nevertheless, the residual stress, the warpage and the replication fidelity of microchannel for the substrate and cover on microfluidic chips should be considered simultaneously to meet the needs of high-performance chips. Few studies have explicitly proposed a detailed method for multi-objective optimization of injection molding on microfluidic chip.

Similar to the optimization of all Microinjection molding products, there are two primary assessment criterions on microfluidic chip molding: the shape accuracy of plate and the molding quality of microchannel, which are in conflict to some extent (the plates of substrate and the cover being formed in the same mold simultaneously). Therefore, it is necessary to establish a balance between them. The optimization of substrate and cover plates for microfluidic chip needs to consider the influence of multiple aspects on the overall performance (the replication fidelity of microchannel, the warpage and the residual stress, etc.), which is a multi-objective optimization problem (MOOP). In the past three decades, scholars have put forward many effective approaches to solve this problem. Normally, there are two main directions employed to deal with the multi-objective problem. One is the global optimization method combining optimization algorithm and surrogate model, named SMOA; the other is the method using a multi-criterial decision method to search the best solution among all sample points, named MCDM (Table 1). To be more specific:

1. In order to obtain the global optimal solution, many optimization algorithms are proposed, such as: genetic algorithm (GA)^19^, non-dominated sorting genetic algorithm (NSGA-II)^20^, multi-objective particle swarm optimization algorithm (MOPSO)^21^, multi-objective genetic algorithm (MOGA)^22^, etc.
2. In order to reduce the experimental workload and improve the optimization efficiency, a large number of surrogate models are adopted. Such as: Kriging model^23^, radial basis function (RBF)^24^, response surface methodology (RSM)^25^, etc.
3. To quickly determine the best compromise, multi-criterial decision making (MCDM) methods are also widely employed. Such as: grey relational analysis (GRA)^26^, technique for order preference by similarity to ideal solution (TOPSIS)^27^, fuzzy logics (FL)^28^ etc.

**Table 1:** The methods of multi-objective optimization.

Optimization direction	Method	Detailedness
SMOA	Surrogate model	Kriging model, RBF, RSM, etc
	Optimization algorithm	GA, NSGA-II, MOPSO, MOGA, etc
MCDM	Multi-criterial decision making	GRA, TOPSIS, FL, etc

According to the literature on multi-objective optimization of injection molding, the multi-objective optimization based on surrogate model and optimization algorithm is the mainstream optimization method at present. However, most of the existing algorithms can work effectively with less than three optimization objectives. In SMOA, when more optimization objectives need to be considered, the application of optimization algorithms will be limited^29,30^. In addition, in order to obtain an accurate surrogate model, a large number of sample points are needed, which is a great challenge to establish an accurate surrogate model. Moreover, because of the strong nonlinearity of the replication fidelity for microchannel on microfluidic chip, the low precision of the established surrogate model will lead to a large deviation between the results of the optimal scheme obtained by the optimization algorithm and the experimental value, which can not ensure the effectiveness of the global optimal scheme (as shown in Table 7). Therefore, surrogate model and optimization algorithm is not suitable for multi-objective optimization in injection molding of microfluidic chip.

In MCDM, the GRA is also widely applied to solve multi-objective optimization problems. Therefore, a method combining GRA and Taguchi design was proposed by researchers^31,32^, which can not only overcome the defect that Taguchi method cannot solve multi-objective optimization^33^, but also mine the information of the whole parameter space with limited sample size as much as possible. However, the GRA cannot provide an optimal solution of highly robust for a given multi-objective optimization problem, because this method cannot quantitatively or qualitatively distinguish the ideal case of foraging problem with no solution (black) and unique solution (white)^34^. In detail, different normalized formulas are applied on the basis of target characteristics, and then the grey relational coefficient and GRG are calculated, which leads to the uncertainty of the optimal solution of GRA^35,36^. Thus, applying GRA method to solve multi-objective optimization problems has certain limitations.

Fuzzy logic was proposed by Zadeh in 1965, which is usually adopted to solve problems of uncertainty and ambiguity^37^. In addition, fuzzy logic has been applied to the field of multi-objective optimization in recent years, because of its applicability, simple and flexible. Tran et al. employed the mothed of grey fuzzy reasoning grade analysis to execute optimization on carbon fiber–reinforced polymer^38^. Yao et al. optimized the beam-like structure by applying the fuzzy decision method^34^. Saini et al. proposed a novel forecast method by combining particle swarm optimization algorithm with fuzzy inference system tree^39^. Shen et al. realized energy management and optimal control by establishing a fuzzy model of automobile fuel cell^40^. At present, the research of multi-objective fuzzy decision making is relatively simple, and there is no definition and guidance for the writing of fuzzy rules. In addition, the existing multi-objective fuzzy decision-making system only applies to the case where the weights of each index are equal, and there is no effective scheme for the case where the weights of each index are different. Therefore, a novel fuzzy decision making method and specific fuzzy rule writing method are proposed to solve the problem of different objective weights and rule generate in multi-objective optimization.

In this paper, the Taguchi design, the grey relational analysis and fuzzy decision method are simultaneously applied to optimize the injection molding process parameters of substrate and cover plates for typical microfluidic chip (protein electrophoresis microfluidic chip).

## Protein electrophoresis microfluidic chip experiment

### Design for protein electrophoresis microfluidic chip

**Figure 1:**
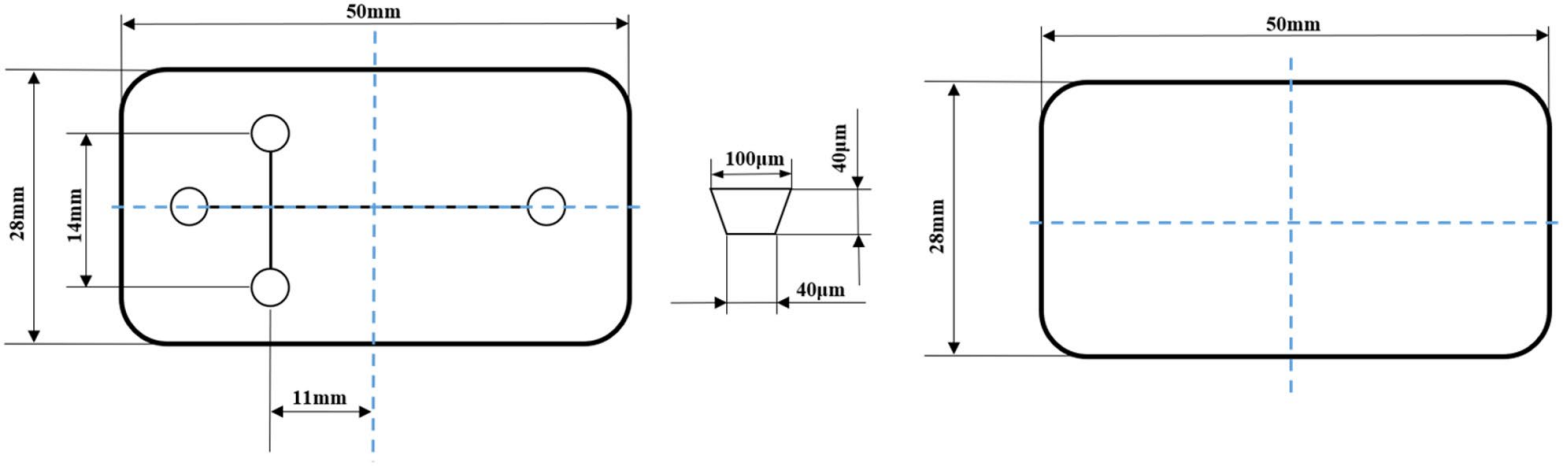
The detailed structure and size of protein electrophoresis microfluidic chip.

In this paper, a cross protein electrophoresis microfluidic chip with a single channel structure was designed. The chip is composed of substrate plate and cover plate, of which the length, width and thickness for the substrate plate are 50 mm, 28 mm and 0.8 mm respectively, while the length, width and thickness for the cover plate are 50 mm, 28 mm and 0.6 mm respectively. The detailed structure and size of chip are shown in Fig. 1.

### Experimental material and equipment

**Figure 2:**
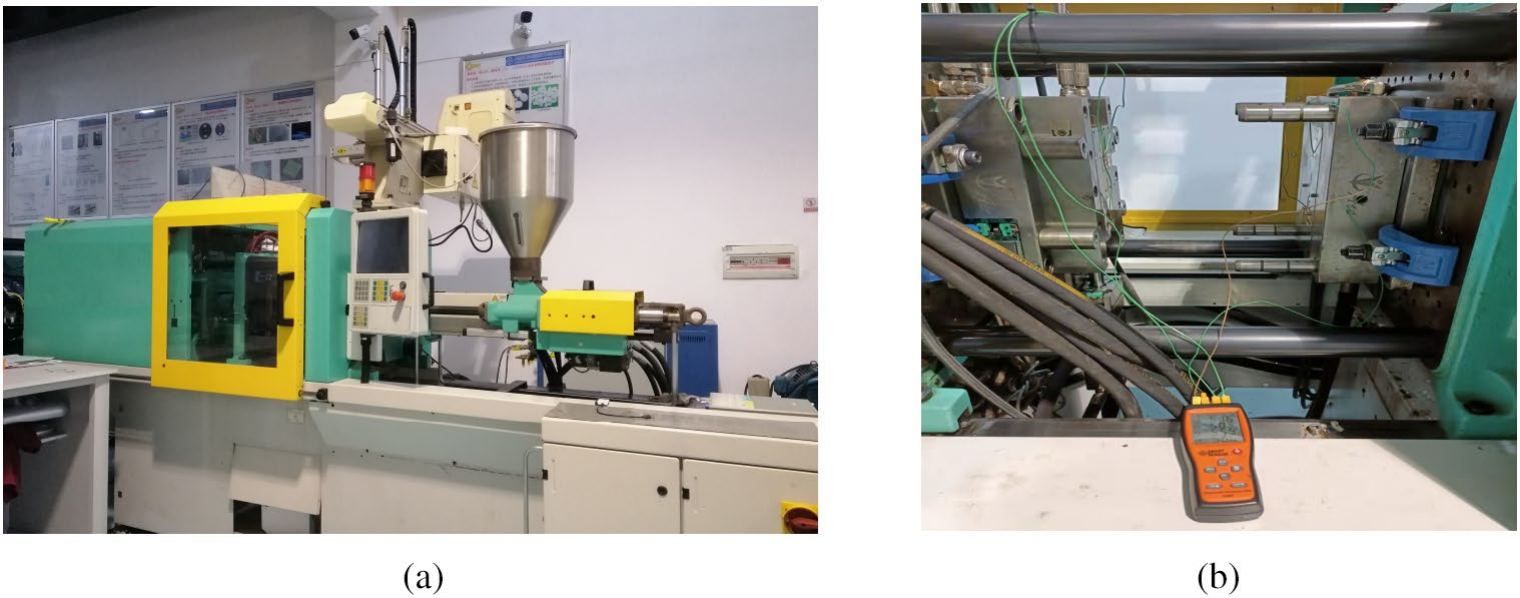
The injection molding system and the mold core of protein electrophoresis microfluidic chips (**a**) The injection molding system (**b**) The mold core.

PMMA was selected as the material for the production of protein electrophoresis microfluidic chips due to its advantages of high light transmittance, good solvent and chemical compatibility in this study. To be more specific, the PMMA CM-205 produced by ChiMei from Taiwan in China was applied, and the properties are shown in Table 2. In addition, the injection molding system (Fig. 2a) includes three parts: the precision injection molding machine, the feeder and the mold temperature machine. This molding system has the advantages of programmable, stable process performance and high temperature control accuracy. The detailed product models, company and production areas are shown in Table 3. Meanwhile, a dual-type cavity mold core that can simultaneously form the substrate and cover plate for the chip was adopted, as shown in Fig. 2b.

**Table 2:** The properties of PMMA CM-205.

Material	Glass-transition temperature (℃)	Density (g/cm^3^)	Coefficient of thermal expansion (/℃)	Poisson ratio	Light transmittance (%)	Biocompatibility
PMMA	105	1.2	0.00005	0.33	90	Better

**Table 3:** The product models and production areas of injection molding system.

Injection molding system	Precision injection molding machine	Feeder	Mold temperature machine
Product models	370S (500–100)	SCD-20U/30H	SIC-3A
Company	ARBURG	SHINI	SHINI
Production areas	Germany	China	China

## The theory of multi-objective optimization for protein electrophoresis microfluidic chip

### The multi-objective optimization method

**Figure 3:**
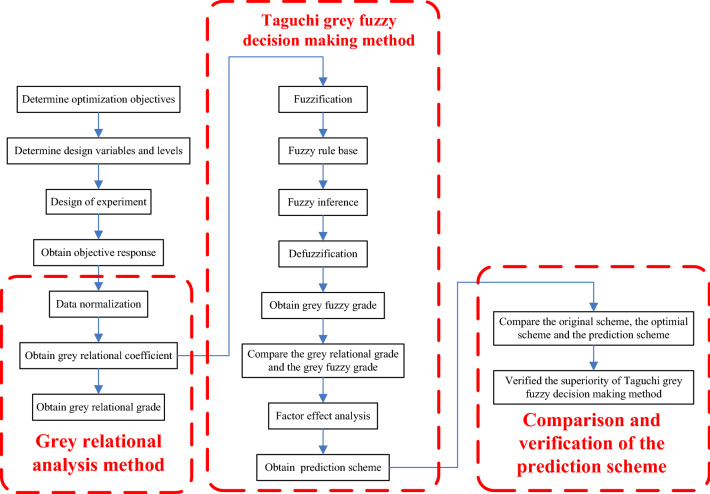
The flow chart of multi-objective optimization method for protein electrophoresis microfluidic chip.

In this paper, the Taguchi orthogonal design method, the grey relational analysis method, the grey Taguchi fuzzy decision method and the factor influence analysis are simultaneously adopted in multi-objective optimization of protein electrophoresis microfluidic chip. Firstly, the design approach for multi-objective optimization is determined and the optimization objectives are determined according to the application requirements of protein electrophoresis microfluidic chip. Secondly, the change level of design variables is determined according to the original design parameters. Then, the orthogonal experimental design is carried out. Thirdly, the response data of optimization objectives is obtained by performing practical experiments. Fourthly, the grey relational analysis method is applied to process the response data to obtain the grey relation grade. In addition, the membership functions for input variables, the membership functions for output variables and corresponding fuzzy subset levels are confirmed. Meanwhile, the fuzzy rules are compiled according to the weights of optimization target. Moreover, the grey fuzzy grade (GFG) was analyzed to predict the optimal combination of process parameters. Finally, the superiority and effectiveness of Taguchi grey fuzzy decision making method are verified by comparing the original scheme, the optimal scheme and the prediction scheme. The flow chart of multi-objective optimization method for protein electrophoresis microfluidic chip is shown in Fig. 3.

### The evaluation criteria of multi-objective optimization

**Figure 4:**
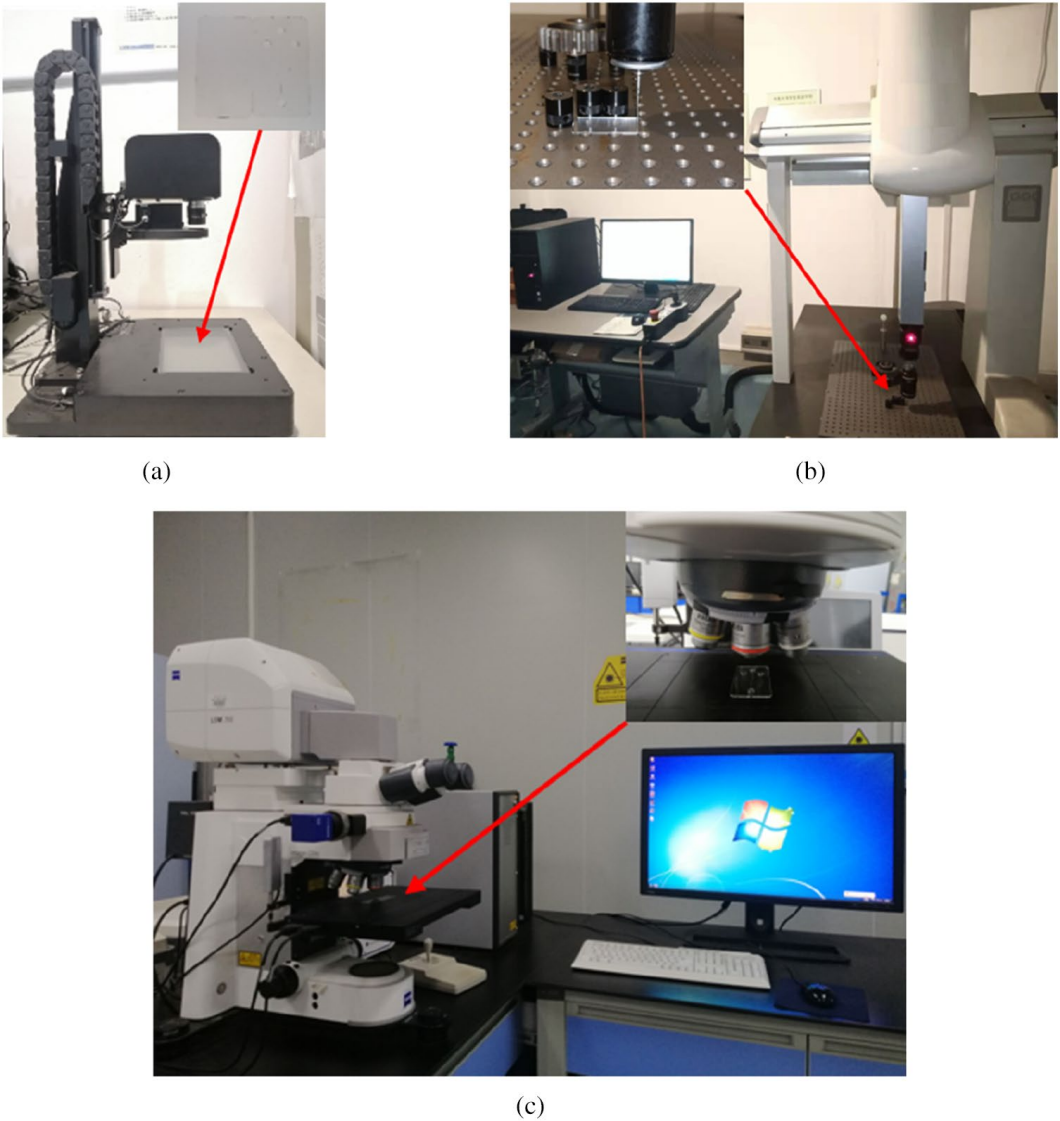
The test equipment for protein electrophoresis microfluidic chip. (**a**) The double refraction instrument. (**b**) The three-coordinate measuring system. (**c**) The laser scanning confocal microscope.

The optimization of protein electrophoresis microfluidic chip has two main evaluation criteria: the shape accuracy of plate and the molding quality of microchannel. To be more specific, the protein electrophoresis microfluidic chip will warp and crack during the storage process because of the residual stress. Hence, the residual stress is one of the shape accuracy criteria. Meanwhile, warpage directly affects the bonding quality of protein electrophoresis microfluidic chips. Therefore, the warpage is one of the shape accuracy criteria. As the main functional unit of protein electrophoresis microfluidic chip, the molding quality of microchannel is particularly important. Accordingly, the replication fidelity is the molding quality of microchannel for evaluation criteria. In particular, the residual stress of the protein electrophoresis microfluidic chip can be characterized by the birefringence effect of the chip. The relationship between refraction value and residual stress can be expressed as $\sigma =\frac{\Delta S}{K\times H}$, where $\sigma$ is the residual stress, $\Delta S$ represents the refraction value, $K$ donates the pressure optical coefficient, $H$ is the thickness of the measured part. The larger the residual stress, the stronger the birefringence effect and the larger the measured value. Likewise, the warpage of the protein electrophoresis microfluidic chip can be characterized by the distance between the maximum value and the minimum value in the vertical direction of the chip surface. In addition, the replication fidelity of microchannel is represented by the mean square root of the ideal microchannel contour and the actual microchannel contour. The formula is as follows^41^:

1 $$MSR=\sqrt{\frac{{({N}_{1}-{n}_{1})}^{2}+{({N}_{2}-{n}_{2})}^{2}+\cdots +{({N}_{i}-{n}_{i})}^{2}}{i}},$$

where $MSR$ represents the value of the mean square root, ${n}_{i}$ is the point of the actual microchannel contour, ${N}_{i}$ denotes the point of the ideal microchannel contour, *i* is the amount of the point for microchannel contour. Table 4 and Fig. 4 show the test equipment and models adopted for residual stress, warpage and replication fidelity of microchannel for protein electrophoresis microfluidic chip.

**Table 4:** The models of test equipment for protein electrophoresis microfluidic chip.

Evaluation criteria	Residual stress	Warpage	Replication fidelity
Equipment	Double refraction instrument	Three coordinate measuring system	Laser scanning confocal microscope
Models	WPA-200	GLOBAL STATUS575	Axio LSM700
Company	Photonic Lattic	Brown & Sharpe Inc	Zeiss
Production areas	Japan	America	Germany

**Figure 5:**
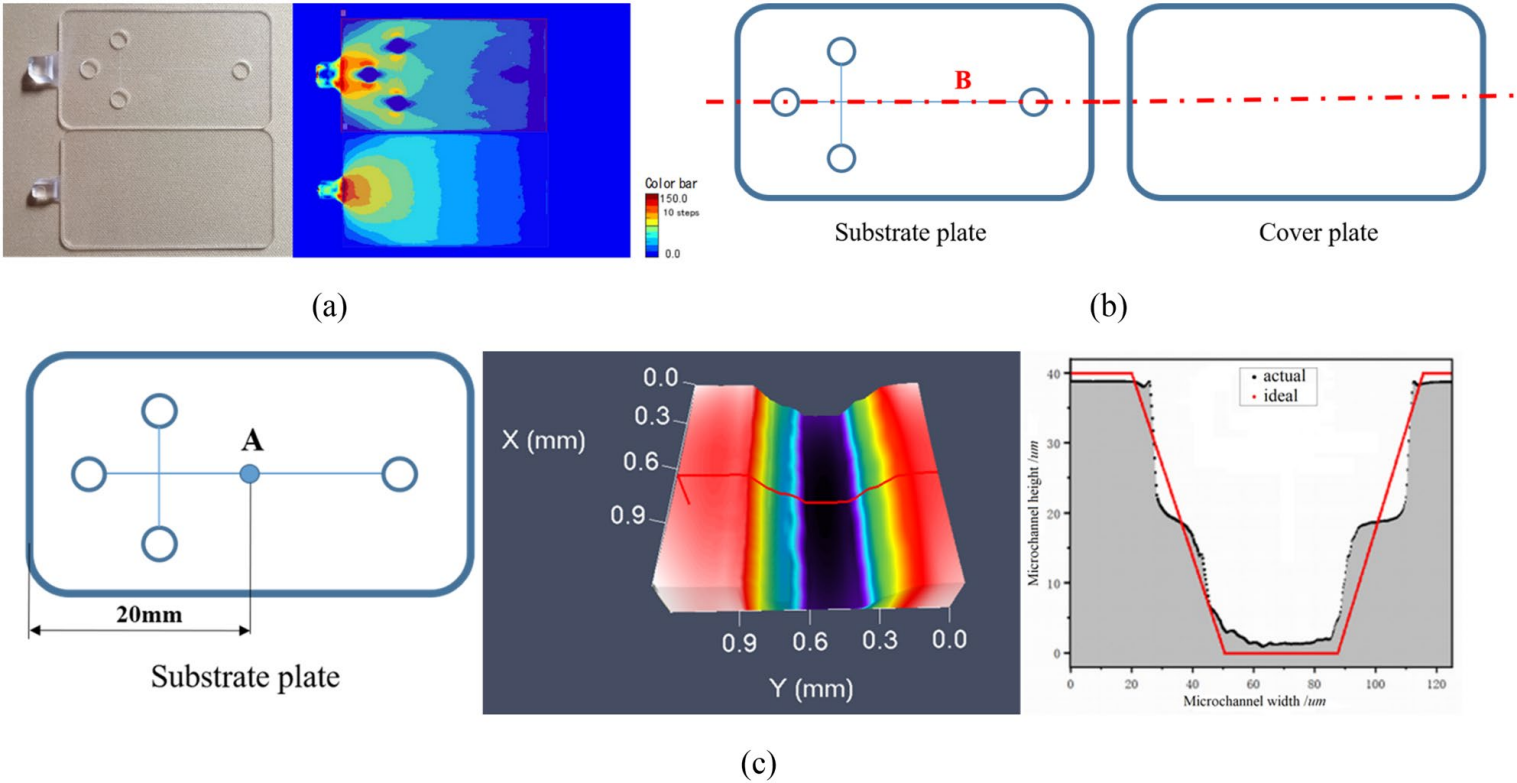
The measurement method of evaluation criteria for protein electrophoresis microfluidic chip. (**a**) The birefringence value. (**b**) The measuring position of warpage. (**c**) The measuring position and detail for replication fidelity of microchannel.

The evaluation criteria for protein electrophoresis microfluidic chip include: (1) the value of residual stress for substrate plate and cover plate, (2) the value of warpage for substrate plate and cover plate, (3) the replication fidelity of microchannel for substrate plate. In this study, the birefringence value for substrate plate and cover plate represents the residual stress for substrate plate and cover plate (Fig. 5a). In Fig. 5a, the substrate plate and cover plate of the microfluidic chip manufactured by injection molding are shown on the left of figure, and the birefringence effect of the substrate plate and cover plate is shown on the right of figure. The distance between the maximum and minimum value of line B in transverse symmetry for substrate and cover plate represents the warpage for substrate plate and cover plate (Fig. 5b). The mean square root of the microchannel at point A, 20 mm from the short edge of the substrate, denotes the replication fidelity of microchannel for substrate plate (Fig. 5c). In Fig. 5c, the positions of microchannels to be measured in the substrate are shown on the left side of the figure. The microchannel morphology of point A measured by laser confocal microscopy is shown in the middle of the figure. The comparison between the actual and ideal microchannel morphology is shown in the right side of the figure.

## The design of experiment design for multi-objective optimization

### Design variables

According to the existing researches on the replication fidelity of microchannel, the warpage and the residual stress of microfluidic chips in injection molding^14,16–18^ and experience in protein electrophoresis microfluidic chip production, five major process parameters were selected as design variables, including the melt temperature (MT), the injection pressure (IP), the injection speed (IS), the packing pressure (PP) and the packing time (PT), in this paper. Five experimental levels are selected for all design variables. The range of process parameters on injection molding for protein electrophoresis microfluidic chip is shown in Table 5.

**Table 5:** The range of process parameters on injection molding for protein electrophoresis microfluidic chip.

Level	MT (°C)	IP (MPa)	IS (cm^3^/s)	PP (MPa)	PT (s)
1	250	150	45	60	4
2	255	160	50	70	5
3	260	170	55	80	6
4	265	180	60	90	7
5	270	190	65	100	8

### The design and results of experiment

An Orthogonal experimental design, L25(5^5^), was carried out for the injection molding process parameters of five factors and five levels in Table 5. Then, according to orthogonal experimental design, the practical experiments are carried out, as shown in Table 6 (each experiment result is the average value of three experiments under the condition of stable process).

**Table 6:** The design and results of experiment.

No.	Design of experiment					Experiment results				
	MT	IP	IS	PP	PT	RSS	RSC	WS	WC	RFM
1	1	5	5	5	5	187.366	191.820	0.325	0.033	5.233
2	2	2	3	4	5	152.551	159.813	0.170	0.049	7.699
3	1	2	2	2	2	111.336	136.645	0.041	0.054	4.846
4	2	4	5	1	2	192.178	200.867	0.807	1.067	4.740
5	3	2	4	1	3	186.764	202.091	0.398	0.885	4.625
6	3	4	1	3	5	182.647	150.300	0.790	0.152	6.900
7	4	2	5	3	1	110.865	126.295	0.800	0.078	10.395
8	4	4	2	5	3	100.503	118.507	0.788	0.162	6.227
9	5	2	1	5	4	110.261	137.909	0.046	0.166	6.151
…	…	…	…	…	…	…	…	…	…	…
25	5	4	3	2	1	174.700	186.004	0.060	1.055	4.925

### Optimization objectives

In the process of protein electrophoresis microfluidic chip optimization, various criteria of substrate plate and cover plate should be considered. In general, the residual stress and the warpage are employed to assess the shape accuracy. In addition, the replication fidelity of microchannel is applied to evaluate the molding quality of microchannel. Hence, five optimization objectives are selected for multi-objective optimization of protein electrophoresis microfluidic chip in this research, which are the residual stress of substrate, the residual stress of cover, the warpage of substrate, the warpage of cover and the replication fidelity of microchannel. The specific objectives are as follows:

1. The value of birefringence for substrate plate;
2. The value of birefringence for cover plate;
3. The distance between the maximum and minimum value of line B in transverse symmetry for substrate plate;
4. The distance between the maximum and minimum value of line B in transverse symmetry for cover plate;
5. The mean square root of the ideal microchannel contour and the actual microchannel contour for the microchannel at point A on the substrate.

## Taguchi grey fuzzy decision making method

### Grey relational analysis method

According to the experimental results of protein electrophoresis microfluidic chip shown in Table 6, the process parameters of design variables MT, IP, IS, PP and PT are taken as the input of the model, while the RSS, RSC, WS, WC and RFM are the output. Meanwhile, different methods were adopted to fit the surrogate models [Kriging model, FRB model, RSM (first order), RSM (second order) and RSM (third order)]. The accuracy (R^2^) of each surrogate model is shown in Table 7. As can be seen from Table 7, the highest accuracy is 0.880 and the lowest accuracy is 0 in the surrogate model. Because of the low accuracy of the established surrogate model, it is not suitable to apply the method of combining surrogate model and optimization algorithm for optimization.

**Table 7:** Accuracy of the surrogate model by using different methods.

Model	Accuracy/R^2^				
	RSS	RSC	WS	WC	RFM
Kriging	0.880	0.726	0.675	0.165	0.343
RBF	0.000	0.355	0.541	0.000	0.000
RSM (first-order)	0.000	0.000	0.000	0.000	0.000
RSM (second-order)	0.210	0.677	0.667	0.458	0.195
RSM (third-order)	0.287	0.677	0.700	0.617	0.296

Grey relational analysis is a multi-index decision making method proposed by Deng in the 1980s^34^. The GRA can transform multi-objective optimization problems into single-objective optimization problems to solve the disadvantages and limitations of Taguchi method that can only deal with single-objective problems. In the data preprocessing stage, different normalization formulas are selected to deal with the original sequences according to the different directions of optimization objective characteristic. To be more specific, on the basis of the characteristics of the target direction "the-bigger-the-better", “the-lower-the-better”, and “the-closer to the target objective value-the-better”, three normalization formulas can be adopted in this approach.

If the optimized direction of original sequence is “the-lower-the-better”, the original sequence can be normalized as follows^30^:

2 $${x}_{i}^{*}\left(t\right)=\frac{{Max}_{t}{x}_{i}\left(t\right)-{x}_{i}\left(t\right)}{{Max}_{t}{x}_{i}\left(t\right)-{Min}_{t}{x}_{i}\left(t\right)}.$$

If the optimized direction for original sequence is “the-bigger-the-better”, the original sequence can be normalized as follows:

3 $${x}_{i}^{*}\left(t\right)=\frac{{x}_{i}\left(t\right)-{Min}_{t}{x}_{i}\left(t\right)}{{Max}_{t}{x}_{i}\left(t\right)-{Min}_{t}{x}_{i}\left(t\right)}.$$

If the optimized direction of original sequence is a target value, namely the feature for original sequence is “the-closer to the target objective value-the-better”, the original sequence can be normalized as follows:

4 $${x}_{i}^{*}\left(t\right)=1-\frac{\left|{x}_{i}\left(t\right)-T\right|}{MAX\left\{{Max}_{t}{x}_{i}\left(t\right)-T , T-{Min}_{t}{x}_{i}\left(t\right)\right\}},$$

where ${x}_{i}^{*}\left(t\right)$ represents the sequence generated by the grey relation, ${x}_{i}\left(t\right)$ denotes the original sequence of experiment results for *i*th element in the sequence of *t*th, ${Max}_{t}{x}_{i}\left(t\right)$ is the maximum value for the sequence of *t*th, ${Min}_{t}{x}_{i}\left(t\right)$ represents the minimum value for the sequence of *t*th, *i* = 1, 2, 3…*n*_1_ and *t* = 1, 2, 3,…*n*_2_, *n*_1_ is the sample size of Taguchi design and *n*_2_ denotes the quantity of optimization objectives, T is the target value.

In this paper, the residual stress of substrate plate, the residual stress of cover plate, the warpage of substrate plate, the warpage of cover plate and the replication fidelity of microchannel should be lowest. Hence, formula (2) is applied to normalize the original sequences of experiment results to [0, 1], as shown in Table 8. Normally, the reference sequence is defined as 1, representing the optimum performances in theory. Therefore, the comparable sequence which is closest to 1 will be considered the optimal scheme.

**Table 8:** The grey relational generation of each objective.

No	Normalization results				
	RSS	RSC	WS	WC	RFM
Ideal	1.000	1.000	1.000	1.000	1.000
1	0.155	0.339	0.657	1.000	0.895
2	0.465	0.628	0.844	0.986	0.467
3	0.832	0.836	1.000	0.981	0.962
4	0.112	0.258	0.076	0.068	0.980
5	0.160	0.246	0.569	0.232	1.000
6	0.197	0.713	0.097	0.893	0.606
7	0.837	0.930	0.084	0.959	0.000
8	0.929	1.000	0.099	0.884	0.722
9	0.842	0.825	0.994	0.880	0.736
…	…	…	…	…	…
25	0.268	0.392	0.977	0.078	0.948

The relation between reference sequence and comparable sequence can be confirmed by grey relational coefficient (GRC). Then, the calculation formula is as follows:

5 $$\upgamma \left({x}_{r}^{*}\left(t\right),{x}_{i}^{*}\left(t\right)\right)=\frac{{\Delta }_{min}+u{\Delta }_{max}}{{\Delta }_{ri}\left(t\right)+u{\Delta }_{max}},$$

where $\gamma \left({x}_{r}^{*}\left(t\right),{x}_{i}^{*}\left(t\right)\right)$ is the grey relational coefficient, *u* denotes the distinguishing coefficient, *u* ∈ [0,1], in general, the value of distinguishing coefficient is 0.5, ${\Delta }_{ri}\left(t\right)$, ${\Delta }_{min}$, ${\Delta }_{max}$ can be deduced by:

6 $${\Delta }_{ri}\left(t\right)=\left|{x}_{r}^{*}\left(t\right)-{x}_{i}^{*}\left(t\right)\right|,$$

7 $$\Delta_{min} = \underbrace {{{\text{min}}}}_{\forall i}\underbrace {{{\text{min}}}}_{\forall t}\Delta_{ri} \left( t \right),$$

8 $$\Delta_{max} = \underbrace {{{\text{max}}}}_{\forall i}\underbrace {{{\text{max}}}}_{\forall t}\Delta_{ri} \left( t \right),$$

Formula (5) is adopted to calculate the normalized results to obtain GRC (Table 8). The grey relational grade (GRG) can be gained by employing GRC. If the weight for each optimization objective is equal, the following formula is used for calculation:

9 $$\mathrm{\varphi }\left({x}_{r}^{*},{x}_{i}^{*}\right)=\frac{1}{n}\sum_{t=1}^{n}\upgamma \left({x}_{r}^{*}\left(t\right),{x}_{i}^{*}\left(t\right)\right).$$

If the weight of each optimization objective is different, GRG can be calculated as follows:

10 $$\mathrm{\varphi }\left({x}_{r}^{*},{x}_{i}^{*}\right)=\sum_{t=1}^{n}{w}_{t}\upgamma \left({x}_{r}^{*}\left(t\right),{x}_{i}^{*}\left(t\right)\right),$$

where *w*_*t*_ denotes the weight of *t*th optimization objective, n represents the amount of the optimization objectives.

In this study, the weights of optimization objectives are different. Hence, GRG is obtained by using formula (10). Meanwhile, the importance of each optimization objective is the replication fidelity of microchannel, the warpage and the residual stress. Moreover, the weights for warpage on the plates of substrate and cover are the same, as is the residual stress for the plates of substrate and cover. The weights of the residual stress of substrate plate, the residual stress of cover plate, the warpage of substrate plate, the warpage of cover plate and the replication fidelity of microchannel are set as 0.1250, 0.1250, 0.1750, 01,750 and 0.4000, respectively (Table 9). As shown in Table 10, the GRGs of experimental results are ranked from big to small, while the No.3 shows the biggest value of GRG in 25 experiments. Therefore, the No.3 is the best scheme for multi-objective optimization of protein electrophoresis microfluidic chip.

**Table 9:** The weights of optimization objectives.

Optimization objectives	RSS	RSC	WS	WC	RFM
Weight	0.1250	0.1250	0.1750	0.1750	0.4000

**Table 10:** The results of grey relational coefficient and grey relational grade.

No	Grey relational coefficient					Grey relational grade	Order
	RSS	RSC	WS	WC	RFM		
Ideal	1.000	1.000	1.000	1.000	1.000		
1	0.372	0.431	0.593	1.000	0.826	0.710	9
2	0.483	0.573	0.763	0.792	0.484	0.629	15
3	0.749	0.754	1.000	0.964	0.929	0.903	1
4	0.360	0.402	0.351	0.349	0.962	0.603	18
5	0.373	0.399	0.537	0.394	1.000	0.660	12
6	0.384	0.636	0.356	0.823	0.559	0.557	22
7	0.754	0.877	0.353	0.925	0.333	0.561	20
8	0.875	1.000	0.357	0.811	0.643	0.696	11
9	0.760	0.741	0.988	0.807	0.654	0.763	5
…	…	…	…	…	…	…	…
25	0.406	0.451	0.956	0.352	0.906	0.698	10

### Grey Fuzzy decision making method

Fuzzy logic, first proposed by Zadeh, is a method to deal with imprecise and undefined boundary problems^36^. Fuzzy decision systems can reason output and calculation results by using fuzzy set theory, and fuzzy sets can provide accurate description by using mathematical expressions.in this study, in order to solve the problems of the uncertainty in GRA optimization results, the fuzzy decision method under different weights and the writing of fuzzy rules, a new fuzzy decision method and specific writing of fuzzy rules are proposed. The new grey fuzzy decision making method can be divided into four steps for analysis and calculation. Meanwhile, the software of MATLAB^®^ is adopted for fuzzy logic analysis in this study.

#### Step 1 Fuzzification

The principle of fuzzification is to apply language variables to transform clear values into fuzzy quantities. Different and appropriate membership functions are selected to assign membership degrees for each language item. There are many membership functions that can be applied, for instance: triangular function, gaussian function, trapezoidal function, γ function and sigmoidal membership function, etc^38^. Among these membership functions, the triangular function was widely adopted due to its simplicity and high calculation efficiency in the existing study^42^. However, in the case of many optimization objectives and high uncertainty of optimization scheme, the selection of triangular function will cause different schemes to have the same fuzzy grade and the order of optimization results is not clear (Table 16). Therefore, the triangular function and the gaussian function are adopted simultaneously to avoid the situation of the same fuzzy grade for output in this study, namely, the gaussian function is employed for input and the triangular function is employed for output. The triangular and gaussian functions are defined as follows^34,37,38^:

11 $$Triangle\left(x,a,b,c\right)=\left\{\begin{array}{l}\begin{array}{ll}0,& x<a\end{array}\\ \begin{array}{ll}\frac{x-a}{b-a},& a\le x\le b\end{array}\\ \begin{array}{l}\begin{array}{ll}\frac{c-x}{c-b},& b<x\le c\end{array}\\ \begin{array}{ll}0,& c<x,\end{array}\end{array}\end{array}\right.$$

12 $$Gaussian\left(x\right)=exp\left[\frac{{-(x-m)}^{2}}{{2\sigma }^{2}}\right], \sigma >0,m>0,$$

where $x$ represents the variable; a, b, and c are the vertices of a triangle; $\sigma$ denotes the coefficient of a gaussian function; m is the center of symmetry of a gaussian function.

**Figure 6:**
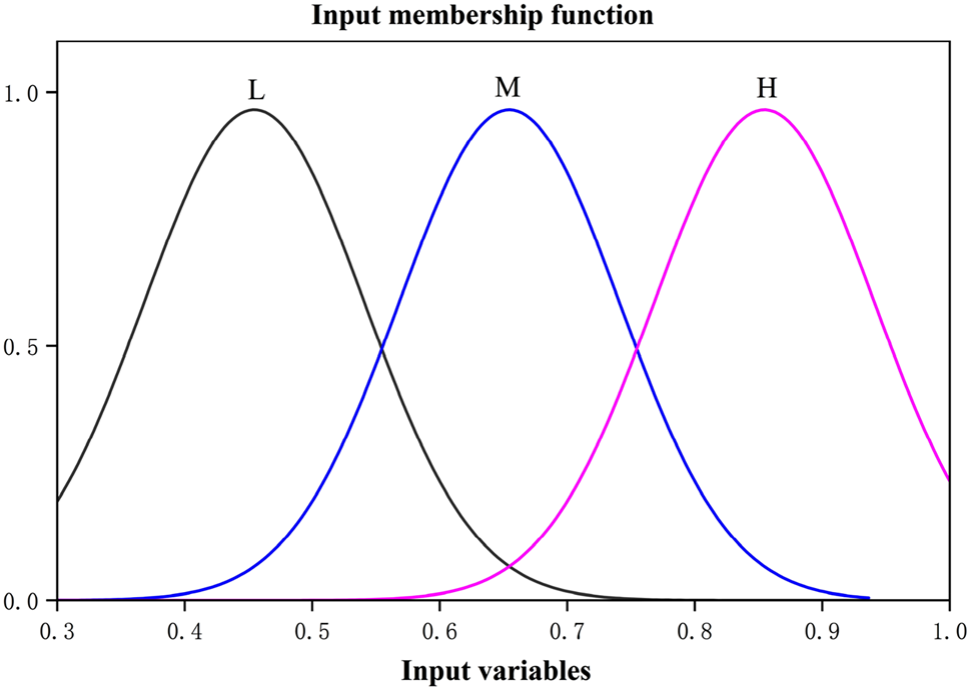
The input membership function.

**Figure 7:**
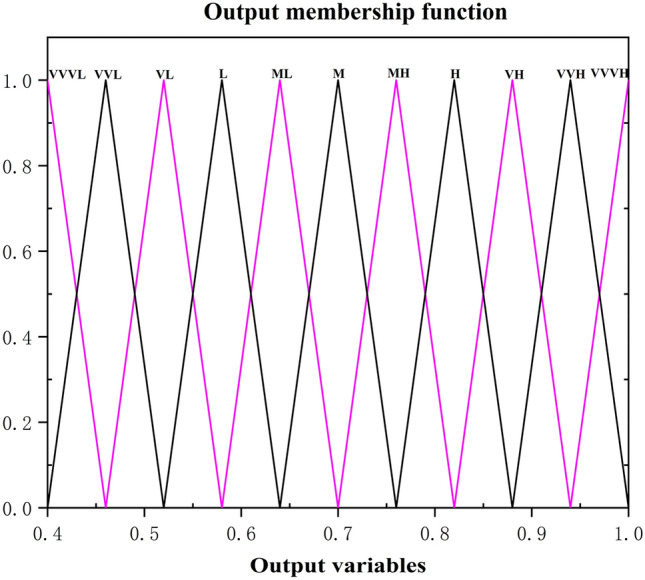
The output membership function.

In this paper, the GRCs of the optimization objectives are adopted as the input variables. Then, the gaussian membership function (12) is applied to fuzzify GRCs into 3 levels: high (H), medium (M) and low (L), namely, the fuzzy subsets of input are divided as 3 grades: H, M and L, as shown in Fig. 6. Correspondingly, the output variables are defined as 11 levels using the triangular membership function (11), which are very very very high (VVVH), very very high (VVH), very high (VH), high (H), between medium and high (MH), medium (M), between medium and low (ML), low(L), very low (VL), very very low (VVL) and very very very low (VVVL) respectively. Likely, the fuzzy subsets of output are divided as 11 grades: VVVL, VVL, VL, H, MH, M, ML, L, VL, VVL and VVVL, as shown in Fig. 7. The value range of fuzzy subset of input and output is shown in Table 11.

**Table 11:** Fuzzy subsets parameter of input and output.

MF	Input		MF	Output	
Gaussian	Level	Value	Triangular	Level	Value
	H	0.07432, 0.825		VVVH	0.94, 1.00, 1.06
	M	0.07432, 0.650		VVH	0.88, 0.94, 1.00
	L	0.07432, 0.475		VH	0.82, 0.88, 0.94
				H	0.76, 0.82, 0.88
				MH	0.70, 0.76, 0.82
				M	0.64, 0.70, 0.76
				ML	0.58, 0.64, 0.70
				L	0.52, 0.58, 0.64
				VL	0.46, 0.52, 0.58
				VVL	0.40, 0.46, 0.52
				VVVL	0.34, 0.40, 0.46

#### Step 2 Fuzzy rule base

Fuzzy rule base is a collection of fuzzy rules, which can be applied to express the relationship between input variables and output variables. In fuzzy decision making, fuzzy rules are the key to perform reasoning. At present, IF–THEN rule is most commonly used and can be expressed as:

$$if\,premise\,\left(antecedent\right), then\,conclusion\,\left(consequent\right).$$

The new and detailed grey fuzzy rules are written as follows: ① Carried out the full-factor design according to the optimization objectives and fuzzy subset grades of the optimization objectives (the fuzzy subset grades of input). ② Divide and match the value 1 according to the grade of the output fuzzy subsets. ③ Redistribute the weight of each optimization objective according to the weight values of the optimization objectives and the fuzzy subset grades of input. ④ Replace The grade of fuzzy subset for each optimization objective in the full-factor design by the corresponding weight value. ⑤ Add the weight values of the same design to obtain the comprehensive weight value, and round the result to one decimal place. ⑥ Obtain the final fuzzy rule scheme by substituting the comprehensive weight value with the corresponding the fuzzy subset grades of input.

To be more specific, during this research, the fuzzy rule base is defined by the criterion that the larger the GRC of each optimization objective is, the higher the GFG value is. the input of fuzzy rule base is composed of five optimization objectives, which are RSS, RSC, WS, WC and RFM respectively. Meanwhile, one output is set, which is GFG. The number of fuzzy subset grades for optimization objectives is 3, and the number of optimization objectives is 5. Therefore, the amount of fuzzy rules should be 243 (3^5^). The full-factor design based on optimization objectives (RSS, RSC, WS, WC and RFM) and fuzzy subset grades of input (H, M and L) is shown in Table 12. The weight values of the fuzzy subset grades on the input for each optimization objective are shown in Table 13. Namely, the weights of H, M and L grades on the fuzzy subset for RSS and RSC are 0.1250, 0.0625 and 0, respectively; the weights of H, M and L grades on the fuzzy subset for WS and WC are 0.1750, 0.0875 and 0, respectively; the weights of H, M and L grades on the fuzzy subset for RFM are 0.4000, 0.2000 and 0, respectively. The values and the fuzzy subset grades of output are shown in Table 14. Namely, the corresponding values the VVVH, VVH, VH, H, MH, M, ML, L, VL, VVL and VVVL grade for the output fuzzy subsets are 1.0, 0.9, 0.8, 0.7, 0.6, 0.5, 0.4, 0.3, 0.2, 0.1 and 0, respectively. The weight values of the grades for each input fuzzy subset, the comprehensive weight value and the grades of corresponding output fuzzy subsets are shown in Table 15 (the “CV” column is the comprehensive weight). The final fuzzy rule scheme of this study is as follows:

**Table 12:** The full-factor design based on optimization objectives and fuzzy subset grades of input.

Full-factor design						Input fuzzy grades replacement				
No	RSS	RSC	WS	WC	RFM	RSS	RSC	WS	WC	RFM
1	1	1	1	1	1	H	H	H	H	H
2	2	2	1	1	1	M	M	H	H	H
3	2	3	1	1	1	M	L	H	H	H
4	3	3	1	2	1	L	L	H	M	H
5	2	3	2	2	1	M	L	M	M	H
6	1	3	3	3	1	H	L	L	L	H
7	3	3	2	2	2	L	L	M	M	M
8	2	2	3	3	2	M	M	L	L	M
9	3	3	2	2	3	L	L	M	M	L
10	2	2	3	3	3	M	M	L	L	L
…	…	…	…	…	…	…	…	…	…	…
243	3	3	3	3	3	L	L	L	L	L

**Table 13:** The weight values of the fuzzy subset grades on the input for each optimization objective.

Optimization objectives	RSS			RSC			WS			WC			RFM		
Weight	0.1250			0.1250			0.1750			0.1750			0.4000		
Fuzzy subset grades of input	H	M	L	H	M	L	H	M	L	H	M	L	H	M	L
Weight	0.1250	0.0625	0	0.1250	0.0625	0	0.1750	0.0875	0	0.1750	0.0875	0	0.4000	0.2000	0

**Table 14:** The values and the fuzzy subset grades of output.

Fuzzy subset grades of output	VVVH	VVH	VH	H	MH	M	ML	L	VL	VVL	VVVL
Value	1.0	0.9	0.8	0.7	0.6	0.5	0.4	0.3	0.2	0.1	0

**Table 15:** The transformation of input fuzzy grade and output fuzzy grade.

Full-factor design and input fuzzy grades replacement						Weight values replacement					Output fuzzy grades replacement	
No	RSS	RSC	WS	WC	RFM	RSS	RSC	WS	WC	RFM	CV	GFG
1	H	H	H	H	H	0.1250	0.1250	0.1750	0.1750	0.4000	1.0	VVVH
2	M	M	H	H	H	0.0625	0.0625	0.1750	0.1750	0.4000	0.9	VVH
3	M	L	H	H	H	0.0625	0.0000	0.1750	0.1750	0.4000	0.8	VH
4	L	L	H	M	H	0.0000	0.0000	0.1750	0.0875	0.4000	0.7	H
5	M	L	M	M	H	0.0625	0.0000	0.0875	0.0875	0.4000	0.6	MH
6	H	L	L	L	H	0.1250	0.0000	0.0000	0.0000	0.4000	0.5	M
7	L	L	M	M	M	0.0000	0.0000	0.0875	0.0875	0.2000	0.4	ML
8	M	M	L	L	M	0.0625	0.0625	0.0000	0.0000	0.2000	0.3	L
9	L	L	M	M	L	0.0000	0.0000	0.0875	0.0875	0.0000	0.2	VL
10	M	M	L	L	L	0.0625	0.0625	0.0000	0.0000	0.0000	0.1	VVL
…	…	…	…	…	…	…	…	…	…	…	…	…
243	L	L	L	L	L	0.0000	0.0000	0.0000	0.0000	0.0000	0	VVVL

$$Rule\,1: if\,"RSS\,is\,H\,and\,RSC\,is\,H\,and\,WS\,is\,H\,and\,WC\,is\,H\,and\,RFM\,is\,H"then\,"GF\, is\,VVVH";else$$

$$Rule\,2: if\,"RSS\,is\,L \,and\,RSC\,is\,L\,and\,WS\,is\,H\,and\,WC\,is\,M\,and\,RFM\,is\,H"then "GFG is H"; else$$

$$\cdots \cdots \cdots \cdots \cdots \cdots \cdots \cdots \cdots \cdots \cdots \cdots \cdots \cdots \cdots \cdots \cdots \cdots \cdots \cdots \cdots \cdots \cdots \cdots$$

13 $$Rule\,243: if\,"RSS\,is\,L\,and\,RSC\,is\,L\,and\,WS\,is\,L\,and\,WC\,is\,L\,and\,RFM\,is\,L"then "GFG is VVVL"$$

#### Step 3 Fuzzy inference

The maximum and minimum operation on Mamdani approach is adopted to execute fuzzy inference for multiple response outputs, and the fuzzy output values are obtained by using fuzzy rule base. The output formula of fuzzy inference is:

14 $${\beta F}_{i}\left({y}_{i}\right)=max\left[\underset{j}{\mathrm{min}}\left\{\beta {A}_{X1}\left({x}_{1}\right), \beta {A}_{X2}\left({x}_{2}\right), \beta {A}_{X3}\left({x}_{3}\right)\cdots \beta {A}_{Xm}\left({x}_{i}\right)\right\}\right],$$

where the ${F}_{i}$ is the fuzzy subsets of output defined by membership functions; ${y}_{i}$ denotes the value of output; ${x}_{i}$ represents the value of input for *m*th optimization objective; $\beta$ is the range of coefficient; ${A}_{Xm}$ denotes the *m*th fuzzy rules; ${\beta F}_{i}\left({y}_{i}\right)$ is the grade of fuzzy inference for output. In this paper, formula (14) is employed to calculate the grade of grey fuzzy inference.

#### Step 4 Defuzzification

The principle of defuzzification is to convert the language output into clear and concrete values. Currently, the center of gravity method can effectively transform fuzzy inference into clear value, which is called fuzzy grade as ${y}^{i}$. The calculation formula is as follows:

15 $${y}^{i}=\frac{\underset{s}{\overset{}{\int }}{y}_{i}\beta {F}_{i}({y}_{i})dy}{\underset{s}{\overset{}{\int }}\beta {F}_{i}({y}_{i})dy}.$$

In this study, (15) is adopted to convert the calculated grades of grey fuzzy inference into clarity values.

## Results and discussion

### Grey fuzzy grade

The GFGs combining the triangular membership function and gaussian membership function, the GFGs of the triangular membership function and the GRGs of the GRA method are listed and arranged from large to small in Table 16. As can be seen from Table 16, some GFGs obtained by adopting triangular membership function are the same, which leads to unclear ordering of optimization results (No. 1, 3, 5 and 8 have the same GFGs). However, this problem does not exist in the combination of triangular membership function and gaussian membership function. Therefore, the GFGs obtained by combining triangular membership function with gaussian membership function has higher accuracy and discrimination.

**Table 16:** The grades and rank by adopting different approaches.

Experiment	Grey relational analysis		Triangular + gaussian		Triangular	
No	GRG	Order	GFG	Order	GFG	Order
1	0.710	9	0.736	10	0.700	10
2	0.629	15	0.677	13	0.653	15
3	0.903	1	0.938	1	0.700	10
4	0.603	18	0.641	16	0.64	17
5	0.660	12	0.673	14	0.700	10
6	0.557	22	0.578	23	0.578	22
7	0.561	20	0.636	18	0.640	17
8	0.696	11	0.721	11	0.700	10
9	0.763	5	0.805	5	0.833	4
…	…	…	…	…	…	…
25	0.698	10	0.759	8	0.760	7

**Figure 8:**
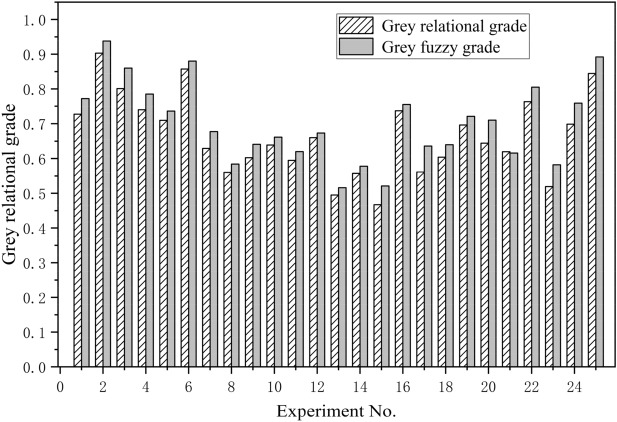
Comparison between the GFGs and the GRGs.

By comparing the GFGs combining the triangular membership function and gaussian membership function with the GRGs of the GRA method, it can be found that the rank of GFGs and GRGs are mostly the same, except for a few schemes with some differences. Meanwhile, compared with the same scheme, the value of GFG is greater than the GRG. Figure 8 makes a more intuitive comparison between the GFGs combining the triangular membership function and the gaussian membership function and the GRGs of the GRA method. The uncertainty of the scheme with grey fuzzy decision method is obviously reduced, and the grade is closer to the ideal value 1. Therefore, the method of Taguchi grey fuzzy decision making can provide a more reliable, qualitative and robust multi-objective optimization platform. In addition, as shown in Table 16, the value of GFG (the triangular membership function and gaussian membership function) and GRG (GRA) of scheme No.3 is the largest. Thus, the No.3 is the optimal compromise among 25 experiments.

### Influence analysis of factors

In Taguchi grey-fuzzy decision making, the average grey fuzzy grade (AGFG) on each design variable for the same level is a significant target to decide the importance on design variables and the optimal parameter combination. The AGFG on each design variable for the same level can be obtained by classification and calculation of each design variable in orthogonal table at the same level. The calculation formula is as follows:

16 $${v}_{ou}=\frac{{N}_{1u}+{N}_{2u}+{N}_{3u}+\cdots +{N}_{ou}}{o},$$

where the ${v}_{ou}$ represents the AGFG of *o*th design variable on *u*th level; ${N}_{ou}$ is the GFG of *o*th design variable on *u*th level; *o* denotes the number of levels; *u* is the number of design variables.

**Figure 9:**
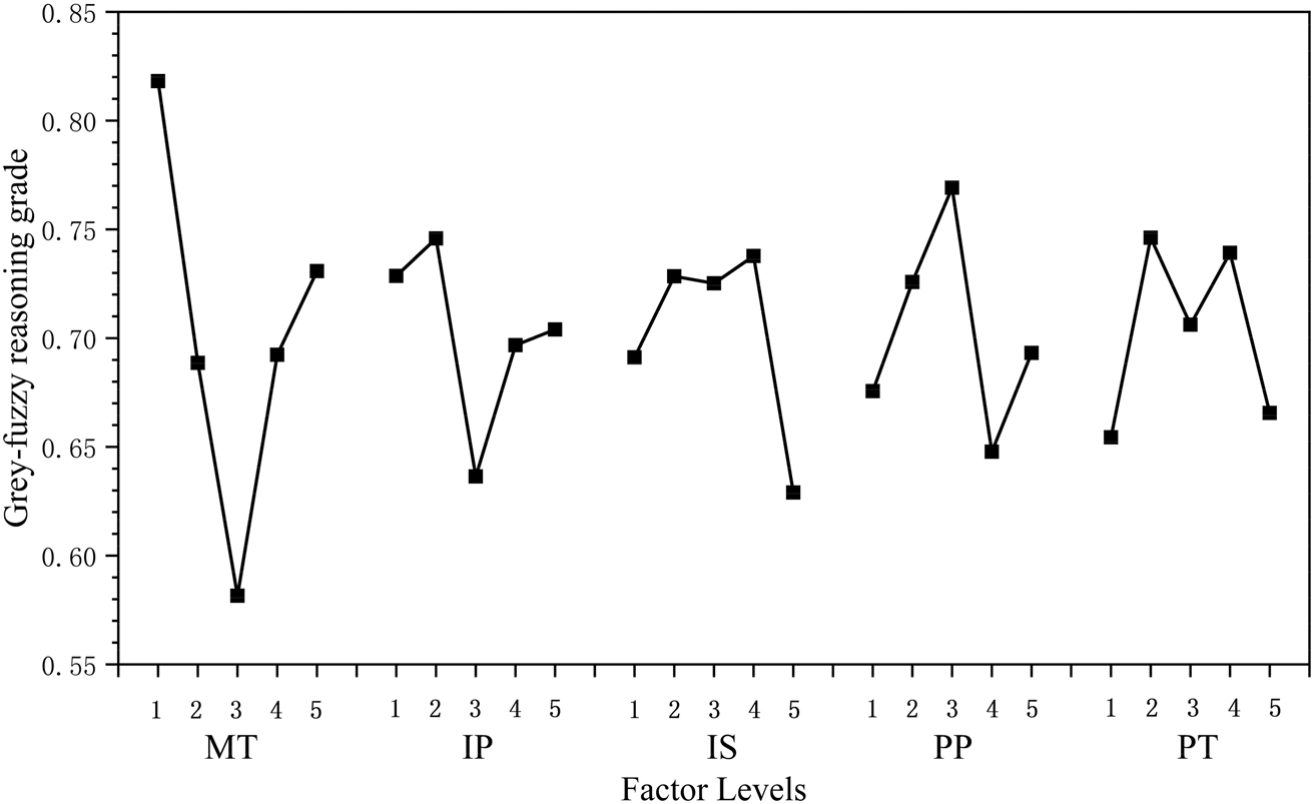
The main effect diagram of factors on GFG.

In this study, formula (16) is employed to classify and calculate the AGFGs (the triangular membership function and the gaussian membership function) in Table 16. The calculation results are shown in Table 17. Moreover, the main effect diagram (Fig. 9) clearly shows the influence of design variables on each optimization objective. It can be seen from Table 17 and Fig. 9 that the maximum values of AGFGs for design variables MT, IP, IS, PP and PT are 0.8182, 0.7458, 0.7378, 0.7692 and 0.7462, respectively. Therefore, the scheme of MT_1_, IP_2_, IS_4_, PP_3_ and PT_2_ (MT: 250 °C, IP:160 MPa, IS: 65 cm^3^/s, PP: 80 MPa and PT: 5 s) is the optimal parameter combination that can simultaneously improve the performance of each optimization objective.

**Table 17:** Main influences of factors for GFGs.

Level	Factor				
	MT	IP	IS	PP	PT
1	0.8182	0.7286	0.6912	0.6756	0.6544
2	0.6886	0.7458	0.7284	0.7258	0.7462
3	0.5816	0.6364	0.7252	0.7692	0.7062
4	0.6924	0.6968	0.7378	0.6478	0.7392
5	0.7308	0.7040	0.6290	0.6932	0.6656
Max–min	0.2366	0.1094	0.1088	0.1214	0.0918

As shown in Table 17, the differences value between the minimum and maximum of AGFGs, arranged from big to small, are the TM, PP, IS, IP and PT. Hence, the design variable (MT) is the most significant to improve the performance of each optimization target for protein electrophoresis microfluidic chip. In addition, all design variables have uncertain effects on the performance of each optimization objective for protein electrophoresis microfluidic chip, according to the slope of GFG curve in Fig. 9. This results also show that the multi-objective optimization of process parameters is necessary to ensure the integrated performance for protein electrophoresis microfluidic chips.

### Optimal scheme prediction and validation

In order to verify the feasibility and performance improvement effect of the optimal scheme for protein electrophoresis microfluidic chip, the GFG of the optimal scheme must be predicted theoretically and tested in practice after determining the optimal scheme of protein electrophoresis microfluidic chip. The prediction formula of GFG is^38^:

17 $${\tau }_{P}=\overline{{\tau }_{p}}+\sum_{i=1}^{u}\left({\tau }_{h}-\overline{{\tau }_{p}}\right),$$

where $\overline{{\tau }_{p}}$ is the total mean value of the AGFGs; ${\tau }_{h}$ denotes the mean value of the best parameter combination in the AGFGs; *u* is the amount for design variables.

**Figure 10:**
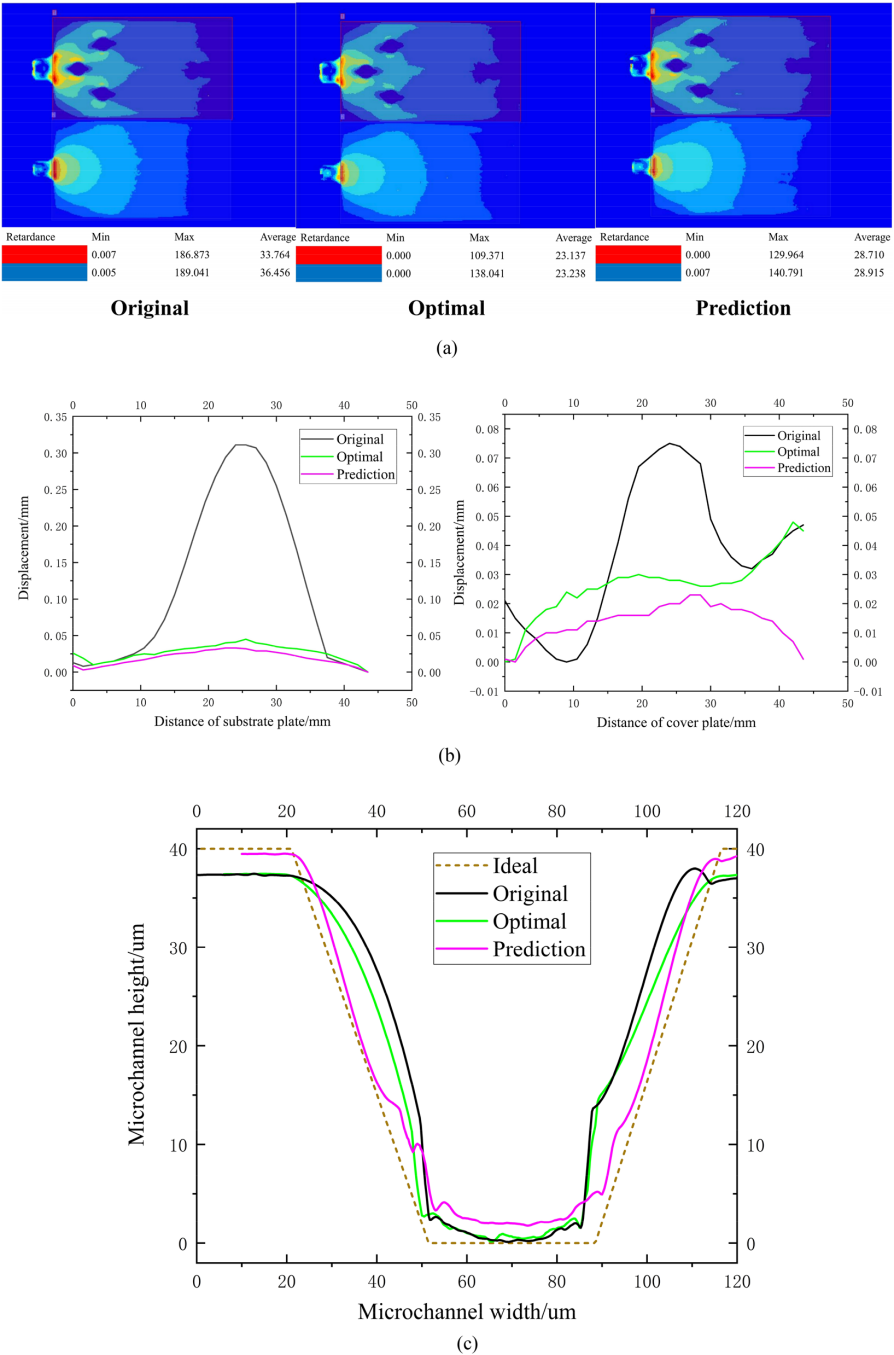
Comparison of the original scheme, the optimal scheme and the prediction scheme in an experiment. (**a**) The residual stress of substrate and cover plate. (**b**) The warpage of substrate and cover plate. (**c**) The replication fidelity of microchannel for substrate plate.

The experimental results and the GFG of the prediction scheme, the optimal scheme and the original scheme are shown in Table 18 (each experiment result is the average value of three experiments under the condition of stable process). Specifically, the “Original” column is the original design, the “Triangular + gaussian” column is the grey fuzzy decision making method applying the triangular membership function and the gaussian membership function, the “Optimal” column is the best scheme obtained by adopting the grey fuzzy decision making method with the triangular membership function and the gaussian membership function, the “Prediction” column is the method of prediction, the “Theory” column is the theory value of prediction method, the “Experiment” column is the experiment value of prediction method, the “Error (%)” column is the relative deviation between the prediction result and the optimal result, the “Improvement (%)” column is the relative deviation between the prediction result and the original design, the “Grey fuzzy grade (new)” column is the grey fuzzy grade obtained by grey fuzzy decision making method after adding the prediction scheme and original design (27 sample points), the “Grey fuzzy grade (old)” column is the GFG obtained by grey fuzzy decision making method (25 sample points). It can be seen from Table 18 that the GFG of the theory prediction (1.008) and the GFG of the experiment prediction (0.902) are differing greatly. The reason for this difference is that after the addition of the prediction scheme and the original scheme, the minimum value of the target sequence is changed, which leads to the change of all the grey relational coefficients and the change of the overall GFGs. (the difference between the Grey fuzzy grade (new) and the grey fuzzy grade (old)). In addition, compared with the original design, the RSS, RSC, WS, WC and RFM of the prediction scheme were reduced by 32.816%, 29.977%, 88.571%, 74.390% and 46.453%, respectively. Compared with the optimal scheme of grey fuzzy decision making method, the RSS, RSC, WS, WC and RFM of the prediction scheme were reduced by − 12.016%, − 5.192%, 12.195%, 61.111% and 30.210%, respectively. Moreover, the GFG of the original design, optimal scheme and prediction scheme is 0.616, 0.874 and 0.902, respectively. The GFGs ranking (high to low) are the prediction scheme, optimal scheme and original design. Figure 10 shows a comparison result of the original scheme, the optimal scheme and the prediction scheme in an experiment. In conclusion, the optimal scheme is the prediction scheme using grey fuzzy decision making method. Accordingly, Taguchi grey fuzzy decision making method can be adopted to optimize protein electrophoresis microfluidic chip effectively.

**Table 18:** Comparison of the original scheme, the optimal scheme and the prediction scheme.

Optimization objective	Triangular + gaussian		Prediction			
	Original	Optimal	Theory	Experiment	Error (%)	Improvement (%)
RSS (nm)	185.704	111.336	–	124.764	12.016	− 32.816
RSC (nm)	188.961	136.645	–	143.654	5.192	− 29.977
WS (mm)	0.315	0.041	–	0.036	− 12.195	− 88.571
WC (mm)	0.082	0.054	–	0.021	− 61.111	− 74.390
RFM	6.316	4.846	–	3.382	− 30.210	− 46.453
Grey fuzzy grade (new)	0.616	0.874	1.008	0.902	3.204	46.429
Grey fuzzy grade (old)	0.658	0.938	–	–	–	–

## Conclusion

During this research, a multi-objective optimization approach and detailed optimization process are proposed for substrate and cover production on protein electrophoresis microfluidic chip, and the effectiveness of the prediction scheme is evaluated through experiments. In more detail, the Taguchi orthogonal design method, the grey relational analysis method, the Taguchi grey fuzzy decision making method and the factor influence analysis are simultaneously adopted in multi-objective optimization of protein electrophoresis microfluidic chip. The main conclusions of this study include:

1. In the optimization of protein electrophoresis microfluidic chip, the grey fuzzy decision making method using the triangular membership function has the problem of insufficient accuracy, which will lead to the same GFG in different schemes, while the grey fuzzy decision method which combines triangular membership function and gaussian membership function can solve this problem and get the appropriate GFG.
2. The detailed fuzzy rule determination method can effectively solve the multi-objective optimization problem with different weights of optimization objectives.
3. Compared with the GRG of the GRA method, the GFG of the grey fuzzy decision making method is closer to the ideal value. The grey fuzzy decision making method can provide a more reliable, qualitative and robust multi-objective optimization platform.
4. The results show that the optimal scheme is achieved when the MT is 250 °C, the IP is 160 MPa, the IS is 65 cm^3^/s, the PP is 80 MPa and the PT is 5 s.
5. Compared with the original design, the RSS, RSC, WS, WC and RFM of the prediction scheme for Taguchi grey fuzzy decision making method were reduced by 32.816%, 29.977%, 88.571%, 74.390% and 46.453%, respectively.

## Supplementary Information

Supplementary Information.

## Data Availability

All data generated or analysed during this study are included in this published article [and its supplementary information files].

## References

[1] Dou M (2019). Rapid and accurate diagnosis of the respiratory disease pertussis on a point-of-care biochip. EClinicalMedicine.

[2] Tavakoli H (2019). Recent advances in microfluidic platforms for single-cell analysis in cancer biology, diagnosis and therapy. Trends Anal. Chem..

[3] Liu Y, Jiang X (2017). Why microfluidics? Merits and trends in chemical synthesis. Lab Chip.

[4] Park E, Lim S (2021). Dynamic phase control with printing and fluidic materials’ interaction by inkjet printing an RF sensor directly on a stereolithographic 3D printed microfluidic structure. Lab Chip.

[5] Raj PM (2021). Fabrication and characterisation of a silicon-borosilicate glass microfluidic device for synchrotron-based hard X-ray spectroscopy studies. RSC Adv..

[6] Jiang S (2021). Numerical simulation and experimental study of the electroosmotic flow in open microfluidic chip based on super-wettability surface. Colloid Interface Sci. Commun..

[7] Ma X (2020). Injection molding and characterization of PMMA-based microfluidic devices. Microsyst. Technol..

[8] Sanjay ST, Dou M, Sun J, Li X (2016). A paper/polymer hybrid microfluidic microplate for rapid quantitative detection of multiple disease biomarkers. Sci. Rep..

[9] Zhou W, Dou M, Timilsina SS, Xu F, Li X (2021). Recent innovations in cost-effective polymer and paper hybrid microfluidic devices. Lab Chip.

[10] Song I-H, Park T (2017). PMMA solution assisted room temperature bonding for PMMA-PC hybrid devices. Micromachines.

[11] Yin Z, Cheng E, Zou H (2018). Fast microfluidic chip fabrication technique by laser erosion and sticky tape assist bonding technique. J. Nanosci. Nanotechnol..

[12] Kim Y (2021). High-throughput injection molded microfluidic device for single-cell analysis of spatiotemporal dynamics. Lab Chip.

[13] Cao Y (2022). Simultaneous detection of multiple foodborne bacteria by loop-mediated isothermal amplification on a microfluidic chip through colorimetric and fluorescent assay. Food Control.

[14] Calaon M (2015). Microfluidic chip designs process optimization and dimensional quality control. Microsyst. Technol..

[15] Xie P, Hu L, He J, Kang W, Yang W (2017). Mechanism and solutions of appearance defects on microfluidic chips manufactured by UV-curing assisted injection molding. J. Polym. Eng..

[16] Jiang, B., Liu, Y., Chu, C. & Qiu, Q. Research on microchannel of PMMA microfluidic chip under various injection molding parameters. In *Advanced Polymer Processing* (eds. Ma, L., Wang, C. & Yang, W.) vols 87–88 381–386 (Trans Tech Publications Ltd, 2010). 10.4028/www.scientific.net/AMR.87-88.381.

[17] Marson S (2011). Flatness optimization of micro-injection moulded parts: The case of a PMMA microfluidic component. J. Micromech. Microeng..

[18] Jena RK, Dev K, Yue CY, Asundi A (2012). Effect of residual stresses in injection molded cyclic olefin copolymer during microfabrication: Hot embossing as well as thermal bonding. RSC Adv..

[19] Kumar D, Dangayach GS, Rao PN (2022). Enhancement of quality of polypropylene by optimisation of injection moulding parameters with genetic algorithm. Int. J. Environ. Sustain. Dev..

[20] Li S, Fan X, Huang H, Cao Y (2020). Multi-objective optimization of injection molding parameters, based on the Gkriging-NSGA-vague method. J. Appl. Polym. Sci..

[21] Zhang J (2016). Multiobjective optimization of injection molding process parameters based on Opt LHD, EBFNN, and MOPSO. Int. J. Adv. Manuf. Technol..

[22] Oktem H, Shinde D (2021). Determination of optimal process parameters for plastic injection molding of polymer materials using multi-objective optimization. J. Mater. Eng. Perform..

[23] Li S (2021). Optimization of injection molding process of transparent complex multi-cavity parts based on Kriging model and various optimization techniques. Arab. J. Sci. Eng..

[24] Heidari BS, Moghaddam AH, Davachi SM, Khamani S, Alihosseini A (2019). Optimization of process parameters in plastic injection molding for minimizing the volumetric shrinkage and warpage using radial basis function (RBF) coupled with the k-fold cross validation technique. J. Polym. Eng..

[25] Kun L, Shilin Y, Yucheng Z, Wenfeng P, Gang Z (2019). Multi-objective optimization of the fiber-reinforced composite injection molding process using Taguchi method, RSM, and NSGA-II. Simul. Model. Pract. Theory.

[26] Kumar, B. P., Venkataramaiah, P. & Ganesh, J. S. Optimization of process parameters in injection moulding of a polymer composite product by using Gra. In *Materials Today-Proceedings*, vol. 18 4637–4647 (Elsevier, 2019).

[27] Ardhiyanto, N. K., Pujiyanto, E. & Rosyidi, C. N. Multi responses optimization of plastic injection molding for biodegradable polymers using Taguchi method and TOPSIS. In *4th International Conference on Industrial, Mechanical, Electrical, and Chemical Engineering* (eds. Anwar, M. *et al*.) vol. 2097 030064 (Amer Inst Physics, 2019). 10.1063/1.5098239.

[28] Ameli K, Alfi A, Aghaebrahimi M (2016). A fuzzy discrete harmony search algorithm applied to annual cost reduction in radial distribution systems. Eng. Optim..

[29] Xiong F (2018). Lightweight optimization of the side structure of automobile body using combined grey relational and principal component analysis. Struct. Multidiscip. Optim..

[30] Shan Z, Long J, Yu P, Shao L, Liao Y (2020). Lightweight optimization of passenger car seat frame based on grey relational analysis and optimized coefficient of variation. Struct. Multidiscip. Optim..

[31] Wang P, Meng P, Zhai J-Y, Zhu Z-Q (2013). A hybrid method using experiment design and grey relational analysis for multiple criteria decision making problems. Knowl. Based Syst..

[32] Wang D, Jiang R, Lu W, Liu H (2016). Optimization of cab suspension parameters of self-dumping trucks using grey relational analysis. J. Grey Syst..

[33] Asokan P, Kumar RR, Jeyapaul R, Santhi M (2008). Development of multi-objective optimization models for electrochemical machining process. Int. J. Adv. Manuf. Technol..

[34] Yao W, Cai K, Xu Y (2021). Optimizing the beam-like structure of a vehicle body using the grey-fuzzy-Taguchi method. Eng. Optim..

[35] Deng J (1989). Introduction to gray system theory. J. Grey Syst..

[36] Elena Arce M, Saavedra A, Miguez JL, Granada E (2015). The use of grey-based methods in multi-criteria decision analysis for the evaluation of sustainable energy systems: A review. Renew. Sustain. Energ. Rev..

[37] Zadeh L (1965). Fuzzy sets. Inf. Comput..

[38] Tran Q-P, Le T-D-M, Huang S-C (2021). Multi-objective optimization of carbon fiber-reinforced polymer drilling process based on grey fuzzy reasoning grade analysis. Int. J. Adv. Manuf. Technol..

[39] Saini J, Dutta M, Marques G (2021). Fuzzy inference system tree with particle swarm optimization and genetic algorithm: A novel approach for PM10 forecasting. Expert. Syst. Appl..

[40] Shen D, Lim C-C, Shi P (2020). Fuzzy model based control for energy management and optimization in fuel cell vehicles. IEEE Trans. Veh. Technol..

[41] Jiang B (2019). Characterization of microchannel replicability of injection molded electrophoresis microfluidic chips. Polymers.

[42] Smaili A, Mrad F (2008). Mechatronics Integrated Technologies for Intelligent Machines.

