# Supplementary material for: A new fuzzy rule based multi-objective optimization method for cross-scale injection molding of protein electrophoresis microfluidic chips
Source: Sci Rep. 2022 Aug 1;12:13159. doi: 10.1038/s41598-022-15935-8 (PMC9343367; doi:10.1038/s41598-022-15935-8)
Supplement: Supplementary file 1 — Supplementary Information. [file 41598_2022_15935_MOESM1_ESM.docx]

**Supplementary Material**

In order to make the table data in the article more comprehensive, we did not select data according to the serial number of the table in the article. Therefore, the experimental serial number in the article does not correspond to the experimental serial number in the supplementary data, but the data in the article can be found in the supplementary data.

**Table S1** The design and results of experiment

| No. | Design of experiment | | | | |  | Experiment results | | | | |
| --- | --- | --- | --- | --- | --- | --- | --- | --- | --- | --- | --- |
|  | MT | IP | IS | PP | PT |  | RSS | RSC | WS | WC | RFM |
| 1 | 1 | 1 | 1 | 1 | 1 |  | 127.644 | 143.084 | 0.203 | 0.034 | 6.125 |
| 2 | 1 | 2 | 2 | 2 | 2 |  | 111.336 | 136.645 | 0.041 | 0.054 | 4.846 |
| 3 | 1 | 3 | 3 | 3 | 3 |  | 141.852 | 154.332 | 0.124 | 0.042 | 5.130 |
| 4 | 1 | 4 | 4 | 4 | 4 |  | 182.027 | 179.531 | 0.119 | 0.038 | 5.444 |
| 5 | 1 | 5 | 5 | 5 | 5 |  | 187.366 | 191.820 | 0.325 | 0.033 | 5.233 |
| 6 | 2 | 1 | 2 | 3 | 4 |  | 140.864 | 157.193 | 0.051 | 0.057 | 4.795 |
| 7 | 2 | 2 | 3 | 4 | 5 |  | 152.551 | 159.813 | 0.170 | 0.049 | 7.699 |
| 8 | 2 | 3 | 4 | 5 | 1 |  | 142.899 | 154.954 | 0.587 | 0.674 | 6.146 |
| 9 | 2 | 4 | 5 | 1 | 2 |  | 192.178 | 200.867 | 0.807 | 1.067 | 4.740 |
| 10 | 2 | 5 | 1 | 2 | 3 |  | 142.285 | 146.453 | 0.576 | 0.085 | 6.295 |
| 11 | 3 | 1 | 3 | 5 | 2 |  | 175.519 | 183.505 | 0.208 | 0.078 | 7.504 |
| 12 | 3 | 2 | 4 | 1 | 3 |  | 186.764 | 202.091 | 0.398 | 0.885 | 4.625 |
| 13 | 3 | 3 | 5 | 2 | 4 |  | 180.617 | 164.867 | 0.807 | 0.154 | 8.402 |
| 14 | 3 | 4 | 1 | 3 | 5 |  | 182.647 | 150.300 | 0.790 | 0.152 | 6.900 |
| 15 | 3 | 5 | 2 | 4 | 1 |  | 204.760 | 139.192 | 0.780 | 1.124 | 7.172 |
| 16 | 4 | 1 | 4 | 2 | 5 |  | 131.122 | 146.740 | 0.299 | 0.782 | 4.640 |
| 17 | 4 | 2 | 5 | 3 | 1 |  | 110.865 | 126.295 | 0.800 | 0.078 | 10.395 |
| 18 | 4 | 3 | 1 | 4 | 2 |  | 100.724 | 127.307 | 0.645 | 0.110 | 8.856 |
| 19 | 4 | 4 | 2 | 5 | 3 |  | 100.503 | 118.507 | 0.788 | 0.162 | 6.227 |
| 20 | 4 | 5 | 3 | 1 | 4 |  | 157.319 | 191.145 | 0.870 | 0.121 | 5.315 |
| 21 | 5 | 1 | 5 | 4 | 3 |  | 92.516 | 124.765 | 0.433 | 0.818 | 6.983 |
| 22 | 5 | 2 | 1 | 5 | 4 |  | 110.261 | 137.909 | 0.046 | 0.166 | 6.151 |
| 23 | 5 | 3 | 2 | 1 | 5 |  | 169.905 | 229.434 | 0.596 | 1.142 | 5.694 |
| 24 | 5 | 4 | 3 | 2 | 1 |  | 174.700 | 186.004 | 0.060 | 1.055 | 4.925 |
| 25 | 5 | 5 | 4 | 3 | 2 |  | 120.342 | 134.194 | 0.120 | 0.044 | 5.086 |

**Table S2** The grey relational generation of each objective

| No. | Experiment results | | | | |  | Normalization results | | | | |
| --- | --- | --- | --- | --- | --- | --- | --- | --- | --- | --- | --- |
|  | RSS | RSC | WS | WC | RFM |  | RSS | RSC | WS | WC | RFM |
| 1 | 127.644 | 143.084 | 0.203 | 0.034 | 6.125 |  | 0.687 | 0.778 | 0.805 | 0.999 | 0.740 |
| 2 | 111.336 | 136.645 | 0.041 | 0.054 | 4.846 |  | 0.832 | 0.836 | 1.000 | 0.981 | 0.962 |
| 3 | 141.852 | 154.332 | 0.124 | 0.042 | 5.130 |  | 0.560 | 0.677 | 0.900 | 0.992 | 0.912 |
| 4 | 182.027 | 179.531 | 0.119 | 0.038 | 5.444 |  | 0.203 | 0.450 | 0.906 | 0.995 | 0.858 |
| 5 | 187.366 | 191.820 | 0.325 | 0.033 | 5.233 |  | 0.155 | 0.339 | 0.657 | 1.000 | 0.895 |
| 6 | 140.864 | 157.193 | 0.051 | 0.057 | 4.795 |  | 0.569 | 0.651 | 0.988 | 0.978 | 0.971 |
| 7 | 152.551 | 159.813 | 0.170 | 0.049 | 7.699 |  | 0.465 | 0.628 | 0.844 | 0.986 | 0.467 |
| 8 | 142.899 | 154.954 | 0.587 | 0.674 | 6.146 |  | 0.551 | 0.671 | 0.341 | 0.422 | 0.736 |
| 9 | 192.178 | 200.867 | 0.807 | 1.067 | 4.740 |  | 0.112 | 0.258 | 0.076 | 0.068 | 0.980 |
| 10 | 142.285 | 146.453 | 0.576 | 0.085 | 6.295 |  | 0.557 | 0.748 | 0.355 | 0.953 | 0.711 |
| 11 | 175.519 | 183.505 | 0.208 | 0.078 | 7.504 |  | 0.261 | 0.414 | 0.799 | 0.959 | 0.501 |
| 12 | 186.764 | 202.091 | 0.398 | 0.885 | 4.625 |  | 0.160 | 0.246 | 0.569 | 0.232 | 1.000 |
| 13 | 180.617 | 164.867 | 0.807 | 0.154 | 8.402 |  | 0.215 | 0.582 | 0.076 | 0.891 | 0.345 |
| 14 | 182.647 | 150.300 | 0.790 | 0.152 | 6.900 |  | 0.197 | 0.713 | 0.097 | 0.893 | 0.606 |
| 15 | 204.760 | 139.192 | 0.780 | 1.124 | 7.172 |  | 0.000 | 0.814 | 0.109 | 0.016 | 0.559 |
| 16 | 131.122 | 146.740 | 0.299 | 0.782 | 4.640 |  | 0.656 | 0.745 | 0.689 | 0.325 | 0.997 |
| 17 | 110.865 | 126.295 | 0.800 | 0.078 | 10.395 |  | 0.837 | 0.930 | 0.084 | 0.959 | 0.000 |
| 18 | 100.724 | 127.307 | 0.645 | 0.110 | 8.856 |  | 0.927 | 0.921 | 0.271 | 0.931 | 0.267 |
| 19 | 100.503 | 118.507 | 0.788 | 0.162 | 6.227 |  | 0.929 | 1.000 | 0.099 | 0.884 | 0.722 |
| 20 | 157.319 | 191.145 | 0.870 | 0.121 | 5.315 |  | 0.423 | 0.345 | 0.000 | 0.921 | 0.880 |
| 21 | 92.516 | 124.765 | 0.433 | 0.818 | 6.983 |  | 1.000 | 0.944 | 0.527 | 0.292 | 0.591 |
| 22 | 110.261 | 137.909 | 0.046 | 0.166 | 6.151 |  | 0.842 | 0.825 | 0.994 | 0.880 | 0.736 |
| 23 | 169.905 | 229.434 | 0.596 | 1.142 | 5.694 |  | 0.311 | 0.000 | 0.331 | 0.000 | 0.815 |
| 24 | 174.700 | 186.004 | 0.060 | 1.055 | 4.925 |  | 0.268 | 0.392 | 0.977 | 0.078 | 0.948 |
| 25 | 120.342 | 134.194 | 0.120 | 0.044 | 5.086 |  | 0.752 | 0.859 | 0.905 | 0.990 | 0.920 |

**Table S3** The results of grey relational coefficient and grey relational grade

| No. | Grey relational coefficient | | | | | Grey relational grade | Order |
| --- | --- | --- | --- | --- | --- | --- | --- |
|  | RSS | RSC | WS | WC | RFM |  |  |
| Ideal | 1.000 | 1.000 | 1.000 | 1.000 | 1.000 |  |  |
| 1 | 0.615 | 0.693 | 0.719 | 0.998 | 0.658 | 0.727 | 8 |
| 2 | 0.749 | 0.754 | 1.000 | 0.964 | 0.929 | 0.903 | 1 |
| 3 | 0.532 | 0.608 | 0.833 | 0.984 | 0.851 | 0.801 | 4 |
| 4 | 0.385 | 0.476 | 0.842 | 0.991 | 0.779 | 0.740 | 6 |
| 5 | 0.372 | 0.431 | 0.593 | 1.000 | 0.826 | 0.710 | 9 |
| 6 | 0.537 | 0.589 | 0.976 | 0.959 | 0.944 | 0.857 | 2 |
| 7 | 0.483 | 0.573 | 0.763 | 0.972 | 0.484 | 0.629 | 15 |
| 8 | 0.527 | 0.603 | 0.432 | 0.464 | 0.655 | 0.560 | 21 |
| 9 | 0.360 | 0.402 | 0.351 | 0.349 | 0.962 | 0.603 | 18 |
| 10 | 0.530 | 0.665 | 0.437 | 0.914 | 0.633 | 0.639 | 14 |
| 11 | 0.403 | 0.460 | 0.713 | 0.925 | 0.501 | 0.595 | 19 |
| 12 | 0.373 | 0.399 | 0.537 | 0.394 | 1.000 | 0.660 | 12 |
| 13 | 0.389 | 0.545 | 0.351 | 0.821 | 0.433 | 0.495 | 24 |
| 14 | 0.384 | 0.636 | 0.356 | 0.823 | 0.559 | 0.557 | 22 |
| 15 | 0.333 | 0.728 | 0.359 | 0.337 | 0.531 | 0.467 | 25 |
| 16 | 0.592 | 0.663 | 0.616 | 0.425 | 0.995 | 0.737 | 7 |
| 17 | 0.754 | 0.877 | 0.353 | 0.925 | 0.333 | 0.561 | 20 |
| 18 | 0.872 | 0.863 | 0.407 | 0.878 | 0.405 | 0.604 | 17 |
| 19 | 0.875 | 1.000 | 0.357 | 0.811 | 0.643 | 0.696 | 11 |
| 20 | 0.464 | 0.433 | 0.333 | 0.863 | 0.807 | 0.644 | 13 |
| 21 | 1.000 | 0.899 | 0.514 | 0.414 | 0.550 | 0.620 | 16 |
| 22 | 0.760 | 0.741 | 0.988 | 0.807 | 0.654 | 0.763 | 5 |
| 23 | 0.420 | 0.333 | 0.428 | 0.333 | 0.730 | 0.519 | 23 |
| 24 | 0.406 | 0.451 | 0.956 | 0.352 | 0.906 | 0.698 | 10 |
| 25 | 0.669 | 0.780 | 0.840 | 0.981 | 0.862 | 0.844 | 3 |

**Table S4** The transformation of input fuzzy grade and output fuzzy grade

| Full-factor design and input fuzzy grades replacement | | | | | |  | Weight values replacement | | | | |  | Output fuzzy grades replacement | |
| --- | --- | --- | --- | --- | --- | --- | --- | --- | --- | --- | --- | --- | --- | --- |
| No. | RSS | RSC | WS | WC | RFM |  | RSS | RSC | WS | WC | RFM |  | CV | GFG |
| 1 | H | H | H | H | H |  | 0.1250 | 0.1250 | 0.1750 | 0.1750 | 0.4000 |  | 1.0 | VVVH |
| 2 | H | M | H | H | H |  | 0.1250 | 0.0625 | 0.1750 | 0.1750 | 0.4000 |  | 0.9 | VVH |
| 3 | H | L | H | H | H |  | 0.1250 | 0.0000 | 0.1750 | 0.1750 | 0.4000 |  | 0.9 | VVH |
| 4 | M | H | H | H | H |  | 0.0625 | 0.1250 | 0.1750 | 0.1750 | 0.4000 |  | 0.9 | VVH |
| 5 | M | M | H | H | H |  | 0.0625 | 0.0625 | 0.1750 | 0.1750 | 0.4000 |  | 0.9 | VVH |
| 7 | L | H | H | H | H |  | 0.0000 | 0.1250 | 0.1750 | 0.1750 | 0.4000 |  | 0.9 | VVH |
| 10 | H | H | H | M | H |  | 0.1250 | 0.1250 | 0.1750 | 0.0875 | 0.4000 |  | 0.9 | VVH |
| 28 | H | H | M | H | H |  | 0.1250 | 0.1250 | 0.0875 | 0.1750 | 0.4000 |  | 0.9 | VVH |
| 6 | M | L | H | H | H |  | 0.0625 | 0.0000 | 0.1750 | 0.1750 | 0.4000 |  | 0.8 | VH |
| 8 | L | M | H | H | H |  | 0.0000 | 0.0625 | 0.1750 | 0.1750 | 0.4000 |  | 0.8 | VH |
| 11 | H | M | H | M | H |  | 0.1250 | 0.0625 | 0.1750 | 0.0875 | 0.4000 |  | 0.9 | VVH |
| 12 | H | L | H | M | H |  | 0.1250 | 0.0000 | 0.1750 | 0.0875 | 0.4000 |  | 0.8 | VH |
| 13 | M | H | H | M | H |  | 0.0625 | 0.1250 | 0.1750 | 0.0875 | 0.4000 |  | 0.9 | VVH |
| 14 | M | M | H | M | H |  | 0.0625 | 0.0625 | 0.1750 | 0.0875 | 0.4000 |  | 0.8 | VH |
| 16 | L | H | H | M | H |  | 0.0000 | 0.1250 | 0.1750 | 0.0875 | 0.4000 |  | 0.8 | VH |
| 19 | H | H | H | L | H |  | 0.1250 | 0.1250 | 0.1750 | 0.0000 | 0.4000 |  | 0.8 | VH |
| 20 | H | M | H | L | H |  | 0.1250 | 0.0625 | 0.1750 | 0.0000 | 0.4000 |  | 0.8 | VH |
| 22 | M | H | H | L | H |  | 0.0625 | 0.1250 | 0.1750 | 0.0000 | 0.4000 |  | 0.8 | VH |
| 29 | H | M | M | H | H |  | 0.1250 | 0.0625 | 0.0875 | 0.1750 | 0.4000 |  | 0.9 | VVH |
| 30 | H | L | M | H | H |  | 0.1250 | 0.0000 | 0.0875 | 0.1750 | 0.4000 |  | 0.8 | VH |
| 31 | M | H | M | H | H |  | 0.0625 | 0.1250 | 0.0875 | 0.1750 | 0.4000 |  | 0.9 | VVH |
| 32 | M | M | M | H | H |  | 0.0625 | 0.0625 | 0.0875 | 0.1750 | 0.4000 |  | 0.8 | VH |
| 34 | L | H | M | H | H |  | 0.0000 | 0.1250 | 0.0875 | 0.1750 | 0.4000 |  | 0.8 | VH |
| 37 | H | H | M | M | H |  | 0.1250 | 0.1250 | 0.0875 | 0.0875 | 0.4000 |  | 0.8 | VH |
| 38 | H | M | M | M | H |  | 0.1250 | 0.0625 | 0.0875 | 0.0875 | 0.4000 |  | 0.8 | VH |
| 40 | M | H | M | M | H |  | 0.0625 | 0.1250 | 0.0875 | 0.0875 | 0.4000 |  | 0.8 | VH |
| 55 | H | H | L | H | H |  | 0.1250 | 0.1250 | 0.0000 | 0.1750 | 0.4000 |  | 0.8 | VH |
| 56 | H | M | L | H | H |  | 0.1250 | 0.0625 | 0.0000 | 0.1750 | 0.4000 |  | 0.8 | VH |
| 58 | M | H | L | H | H |  | 0.0625 | 0.1250 | 0.0000 | 0.1750 | 0.4000 |  | 0.8 | VH |
| 82 | H | H | H | H | M |  | 0.1250 | 0.1250 | 0.1750 | 0.1750 | 0.2000 |  | 0.8 | VH |
| 9 | L | L | H | H | H |  | 0.0000 | 0.0000 | 0.1750 | 0.1750 | 0.4000 |  | 0.8 | VH |
| 15 | M | L | H | M | H |  | 0.0625 | 0.0000 | 0.1750 | 0.0875 | 0.4000 |  | 0.7 | H |
| 17 | L | M | H | M | H |  | 0.0000 | 0.0625 | 0.1750 | 0.0875 | 0.4000 |  | 0.7 | H |
| 18 | L | L | H | M | H |  | 0.0000 | 0.0000 | 0.1750 | 0.0875 | 0.4000 |  | 0.7 | H |
| 21 | H | L | H | L | H |  | 0.1250 | 0.0000 | 0.1750 | 0.0000 | 0.4000 |  | 0.7 | H |
| 23 | M | M | H | L | H |  | 0.0625 | 0.0625 | 0.1750 | 0.0000 | 0.4000 |  | 0.7 | H |
| 25 | L | H | H | L | H |  | 0.0000 | 0.1250 | 0.1750 | 0.0000 | 0.4000 |  | 0.7 | H |
| 33 | M | L | M | H | H |  | 0.0625 | 0.0000 | 0.0875 | 0.1750 | 0.4000 |  | 0.7 | H |
| 35 | L | M | M | H | H |  | 0.0000 | 0.0625 | 0.0875 | 0.1750 | 0.4000 |  | 0.7 | H |
| 36 | L | L | M | H | H |  | 0.0000 | 0.0000 | 0.0875 | 0.1750 | 0.4000 |  | 0.7 | H |
| 39 | H | L | M | M | H |  | 0.1250 | 0.0000 | 0.0875 | 0.0875 | 0.4000 |  | 0.7 | H |
| 41 | M | M | M | M | H |  | 0.0625 | 0.0625 | 0.0875 | 0.0875 | 0.4000 |  | 0.7 | H |
| 43 | L | H | M | M | H |  | 0.0000 | 0.1250 | 0.0875 | 0.0875 | 0.4000 |  | 0.7 | H |
| 46 | H | H | M | L | H |  | 0.1250 | 0.1250 | 0.0875 | 0.0000 | 0.4000 |  | 0.7 | H |
| 47 | H | M | M | L | H |  | 0.1250 | 0.0625 | 0.0875 | 0.0000 | 0.4000 |  | 0.7 | H |
| 49 | M | H | M | L | H |  | 0.0625 | 0.1250 | 0.0875 | 0.0000 | 0.4000 |  | 0.7 | H |
| 57 | H | L | L | H | H |  | 0.1250 | 0.0000 | 0.0000 | 0.1750 | 0.4000 |  | 0.7 | H |
| 59 | M | M | L | H | H |  | 0.0625 | 0.0625 | 0.0000 | 0.1750 | 0.4000 |  | 0.7 | H |
| 61 | L | H | L | H | H |  | 0.0000 | 0.1250 | 0.0000 | 0.1750 | 0.4000 |  | 0.7 | H |
| 64 | H | H | L | M | H |  | 0.1250 | 0.1250 | 0.0000 | 0.0875 | 0.4000 |  | 0.7 | H |
| 65 | H | M | L | M | H |  | 0.1250 | 0.0625 | 0.0000 | 0.0875 | 0.4000 |  | 0.7 | H |
| 67 | M | H | L | M | H |  | 0.0625 | 0.1250 | 0.0000 | 0.0875 | 0.4000 |  | 0.7 | H |
| 83 | H | M | H | H | M |  | 0.1250 | 0.0625 | 0.1750 | 0.1750 | 0.2000 |  | 0.7 | H |
| 84 | H | L | H | H | M |  | 0.1250 | 0.0000 | 0.1750 | 0.1750 | 0.2000 |  | 0.7 | H |
| 85 | M | H | H | H | M |  | 0.0625 | 0.1250 | 0.1750 | 0.1750 | 0.2000 |  | 0.7 | H |
| 86 | M | M | H | H | M |  | 0.0625 | 0.0625 | 0.1750 | 0.1750 | 0.2000 |  | 0.7 | H |
| 88 | L | H | H | H | M |  | 0.0000 | 0.1250 | 0.1750 | 0.1750 | 0.2000 |  | 0.7 | H |
| 91 | H | H | H | M | M |  | 0.1250 | 0.1250 | 0.1750 | 0.0875 | 0.2000 |  | 0.7 | H |
| 109 | H | H | M | H | M |  | 0.1250 | 0.1250 | 0.0875 | 0.1750 | 0.2000 |  | 0.7 | H |
| 24 | M | L | H | L | H |  | 0.0625 | 0.0000 | 0.1750 | 0.0000 | 0.4000 |  | 0.6 | MH |
| 26 | L | M | H | L | H |  | 0.0000 | 0.0625 | 0.1750 | 0.0000 | 0.4000 |  | 0.6 | MH |
| 27 | L | L | H | L | H |  | 0.0000 | 0.0000 | 0.1750 | 0.0000 | 0.4000 |  | 0.6 | MH |
| 42 | M | L | M | M | H |  | 0.0625 | 0.0000 | 0.0875 | 0.0875 | 0.4000 |  | 0.6 | MH |
| 44 | L | M | M | M | H |  | 0.0000 | 0.0625 | 0.0875 | 0.0875 | 0.4000 |  | 0.6 | MH |
| 45 | L | L | M | M | H |  | 0.0000 | 0.0000 | 0.0875 | 0.0875 | 0.4000 |  | 0.6 | MH |
| 48 | H | L | M | L | H |  | 0.1250 | 0.0000 | 0.0875 | 0.0000 | 0.4000 |  | 0.6 | MH |
| 50 | M | M | M | L | H |  | 0.0625 | 0.0625 | 0.0875 | 0.0000 | 0.4000 |  | 0.6 | MH |
| 52 | L | H | M | L | H |  | 0.0000 | 0.1250 | 0.0875 | 0.0000 | 0.4000 |  | 0.6 | MH |
| 60 | M | L | L | H | H |  | 0.0625 | 0.0000 | 0.0000 | 0.1750 | 0.4000 |  | 0.6 | MH |
| 62 | L | M | L | H | H |  | 0.0000 | 0.0625 | 0.0000 | 0.1750 | 0.4000 |  | 0.6 | MH |
| 63 | L | L | L | H | H |  | 0.0000 | 0.0000 | 0.0000 | 0.1750 | 0.4000 |  | 0.6 | MH |
| 66 | H | L | L | M | H |  | 0.1250 | 0.0000 | 0.0000 | 0.0875 | 0.4000 |  | 0.6 | MH |
| 68 | M | M | L | M | H |  | 0.0625 | 0.0625 | 0.0000 | 0.0875 | 0.4000 |  | 0.6 | MH |
| 70 | L | H | L | M | H |  | 0.0000 | 0.1250 | 0.0000 | 0.0875 | 0.4000 |  | 0.6 | MH |
| 73 | H | H | L | L | H |  | 0.1250 | 0.1250 | 0.0000 | 0.0000 | 0.4000 |  | 0.7 | H |
| 74 | H | M | L | L | H |  | 0.1250 | 0.0625 | 0.0000 | 0.0000 | 0.4000 |  | 0.6 | MH |
| 76 | M | H | L | L | H |  | 0.0625 | 0.1250 | 0.0000 | 0.0000 | 0.4000 |  | 0.6 | MH |
| 87 | M | L | H | H | M |  | 0.0625 | 0.0000 | 0.1750 | 0.1750 | 0.2000 |  | 0.6 | MH |
| 89 | L | M | H | H | M |  | 0.0000 | 0.0625 | 0.1750 | 0.1750 | 0.2000 |  | 0.6 | MH |
| 92 | H | M | H | M | M |  | 0.1250 | 0.0625 | 0.1750 | 0.0875 | 0.2000 |  | 0.7 | H |
| 93 | H | L | H | M | M |  | 0.1250 | 0.0000 | 0.1750 | 0.0875 | 0.2000 |  | 0.6 | MH |
| 94 | M | H | H | M | M |  | 0.0625 | 0.1250 | 0.1750 | 0.0875 | 0.2000 |  | 0.7 | H |
| 95 | M | M | H | M | M |  | 0.0625 | 0.0625 | 0.1750 | 0.0875 | 0.2000 |  | 0.6 | MH |
| 97 | L | H | H | M | M |  | 0.0000 | 0.1250 | 0.1750 | 0.0875 | 0.2000 |  | 0.6 | MH |
| 100 | H | H | H | L | M |  | 0.1250 | 0.1250 | 0.1750 | 0.0000 | 0.2000 |  | 0.6 | MH |
| 101 | H | M | H | L | M |  | 0.1250 | 0.0625 | 0.1750 | 0.0000 | 0.2000 |  | 0.6 | MH |
| 103 | M | H | H | L | M |  | 0.0625 | 0.1250 | 0.1750 | 0.0000 | 0.2000 |  | 0.6 | MH |
| 110 | H | M | M | H | M |  | 0.1250 | 0.0625 | 0.0875 | 0.1750 | 0.2000 |  | 0.7 | H |
| 111 | H | L | M | H | M |  | 0.1250 | 0.0000 | 0.0875 | 0.1750 | 0.2000 |  | 0.6 | MH |
| 112 | M | H | M | H | M |  | 0.0625 | 0.1250 | 0.0875 | 0.1750 | 0.2000 |  | 0.7 | H |
| 113 | M | M | M | H | M |  | 0.0625 | 0.0625 | 0.0875 | 0.1750 | 0.2000 |  | 0.6 | MH |
| 115 | L | H | M | H | M |  | 0.0000 | 0.1250 | 0.0875 | 0.1750 | 0.2000 |  | 0.6 | MH |
| 118 | H | H | M | M | M |  | 0.1250 | 0.1250 | 0.0875 | 0.0875 | 0.2000 |  | 0.6 | MH |
| 119 | H | M | M | M | M |  | 0.1250 | 0.0625 | 0.0875 | 0.0875 | 0.2000 |  | 0.6 | MH |
| 121 | M | H | M | M | M |  | 0.0625 | 0.1250 | 0.0875 | 0.0875 | 0.2000 |  | 0.6 | MH |
| 136 | H | H | L | H | M |  | 0.1250 | 0.1250 | 0.0000 | 0.1750 | 0.2000 |  | 0.6 | MH |
| 137 | H | M | L | H | M |  | 0.1250 | 0.0625 | 0.0000 | 0.1750 | 0.2000 |  | 0.6 | MH |
| 139 | M | H | L | H | M |  | 0.0625 | 0.1250 | 0.0000 | 0.1750 | 0.2000 |  | 0.6 | MH |
| 163 | H | H | H | H | L |  | 0.1250 | 0.1250 | 0.1750 | 0.1750 | 0.0000 |  | 0.6 | MH |
| 51 | M | L | M | L | H |  | 0.0625 | 0.0000 | 0.0875 | 0.0000 | 0.4000 |  | 0.6 | MH |
| 53 | L | M | M | L | H |  | 0.0000 | 0.0625 | 0.0875 | 0.0000 | 0.4000 |  | 0.6 | MH |
| 54 | L | L | M | L | H |  | 0.0000 | 0.0000 | 0.0875 | 0.0000 | 0.4000 |  | 0.5 | M |
| 69 | M | L | L | M | H |  | 0.0625 | 0.0000 | 0.0000 | 0.0875 | 0.4000 |  | 0.6 | MH |
| 71 | L | M | L | M | H |  | 0.0000 | 0.0625 | 0.0000 | 0.0875 | 0.4000 |  | 0.6 | MH |
| 72 | L | L | L | M | H |  | 0.0000 | 0.0000 | 0.0000 | 0.0875 | 0.4000 |  | 0.5 | M |
| 75 | H | L | L | L | H |  | 0.1250 | 0.0000 | 0.0000 | 0.0000 | 0.4000 |  | 0.5 | M |
| 77 | M | M | L | L | H |  | 0.0625 | 0.0625 | 0.0000 | 0.0000 | 0.4000 |  | 0.5 | M |
| 78 | M | L | L | L | H |  | 0.0625 | 0.0000 | 0.0000 | 0.0000 | 0.4000 |  | 0.5 | M |
| 79 | L | H | L | L | H |  | 0.0000 | 0.1250 | 0.0000 | 0.0000 | 0.4000 |  | 0.5 | M |
| 80 | L | M | L | L | H |  | 0.0000 | 0.0625 | 0.0000 | 0.0000 | 0.4000 |  | 0.5 | M |
| 90 | L | L | H | H | M |  | 0.0000 | 0.0000 | 0.1750 | 0.1750 | 0.2000 |  | 0.6 | MH |
| 96 | M | L | H | M | M |  | 0.0625 | 0.0000 | 0.1750 | 0.0875 | 0.2000 |  | 0.5 | M |
| 98 | L | M | H | M | M |  | 0.0000 | 0.0625 | 0.1750 | 0.0875 | 0.2000 |  | 0.5 | M |
| 99 | L | L | H | M | M |  | 0.0000 | 0.0000 | 0.1750 | 0.0875 | 0.2000 |  | 0.5 | M |
| 102 | H | L | H | L | M |  | 0.1250 | 0.0000 | 0.1750 | 0.0000 | 0.2000 |  | 0.5 | M |
| 104 | M | M | H | L | M |  | 0.0625 | 0.0625 | 0.1750 | 0.0000 | 0.2000 |  | 0.5 | M |
| 106 | L | H | H | L | M |  | 0.0000 | 0.1250 | 0.1750 | 0.0000 | 0.2000 |  | 0.5 | M |
| 114 | M | L | M | H | M |  | 0.0625 | 0.0000 | 0.0875 | 0.1750 | 0.2000 |  | 0.5 | M |
| 116 | L | M | M | H | M |  | 0.0000 | 0.0625 | 0.0875 | 0.1750 | 0.2000 |  | 0.5 | M |
| 117 | L | L | M | H | M |  | 0.0000 | 0.0000 | 0.0875 | 0.1750 | 0.2000 |  | 0.5 | M |
| 120 | H | L | M | M | M |  | 0.1250 | 0.0000 | 0.0875 | 0.0875 | 0.2000 |  | 0.5 | M |
| 122 | M | M | M | M | M |  | 0.0625 | 0.0625 | 0.0875 | 0.0875 | 0.2000 |  | 0.5 | M |
| 124 | L | H | M | M | M |  | 0.0000 | 0.1250 | 0.0875 | 0.0875 | 0.2000 |  | 0.5 | M |
| 127 | H | H | M | L | M |  | 0.1250 | 0.1250 | 0.0875 | 0.0000 | 0.2000 |  | 0.5 | M |
| 128 | H | M | M | L | M |  | 0.1250 | 0.0625 | 0.0875 | 0.0000 | 0.2000 |  | 0.5 | M |
| 130 | M | H | M | L | M |  | 0.0625 | 0.1250 | 0.0875 | 0.0000 | 0.2000 |  | 0.5 | M |
| 138 | H | L | L | H | M |  | 0.1250 | 0.0000 | 0.0000 | 0.1750 | 0.2000 |  | 0.5 | M |
| 140 | M | M | L | H | M |  | 0.0625 | 0.0625 | 0.0000 | 0.1750 | 0.2000 |  | 0.5 | M |
| 142 | L | H | L | H | M |  | 0.0000 | 0.1250 | 0.0000 | 0.1750 | 0.2000 |  | 0.5 | M |
| 145 | H | H | L | M | M |  | 0.1250 | 0.1250 | 0.0000 | 0.0875 | 0.2000 |  | 0.5 | M |
| 146 | H | M | L | M | M |  | 0.1250 | 0.0625 | 0.0000 | 0.0875 | 0.2000 |  | 0.5 | M |
| 148 | M | H | L | M | M |  | 0.0625 | 0.1250 | 0.0000 | 0.0875 | 0.2000 |  | 0.5 | M |
| 164 | H | M | H | H | L |  | 0.1250 | 0.0625 | 0.1750 | 0.1750 | 0.0000 |  | 0.5 | M |
| 165 | H | L | H | H | L |  | 0.1250 | 0.0000 | 0.1750 | 0.1750 | 0.0000 |  | 0.5 | M |
| 166 | M | H | H | H | L |  | 0.0625 | 0.1250 | 0.1750 | 0.1750 | 0.0000 |  | 0.5 | M |
| 167 | M | M | H | H | L |  | 0.0625 | 0.0625 | 0.1750 | 0.1750 | 0.0000 |  | 0.5 | M |
| 169 | L | H | H | H | L |  | 0.0000 | 0.1250 | 0.1750 | 0.1750 | 0.0000 |  | 0.5 | M |
| 172 | H | H | H | M | L |  | 0.1250 | 0.1250 | 0.1750 | 0.0875 | 0.0000 |  | 0.5 | M |
| 190 | H | H | M | H | L |  | 0.1250 | 0.1250 | 0.0875 | 0.1750 | 0.0000 |  | 0.5 | M |
| 81 | L | L | L | L | H |  | 0.0000 | 0.0000 | 0.0000 | 0.0000 | 0.4000 |  | 0.4 | ML |
| 105 | M | L | H | L | M |  | 0.0625 | 0.0000 | 0.1750 | 0.0000 | 0.2000 |  | 0.4 | ML |
| 107 | L | M | H | L | M |  | 0.0000 | 0.0625 | 0.1750 | 0.0000 | 0.2000 |  | 0.4 | ML |
| 108 | L | L | H | L | M |  | 0.0000 | 0.0000 | 0.1750 | 0.0000 | 0.2000 |  | 0.4 | ML |
| 123 | M | L | M | M | M |  | 0.0625 | 0.0000 | 0.0875 | 0.0875 | 0.2000 |  | 0.4 | ML |
| 125 | L | M | M | M | M |  | 0.0000 | 0.0625 | 0.0875 | 0.0875 | 0.2000 |  | 0.4 | ML |
| 126 | L | L | M | M | M |  | 0.0000 | 0.0000 | 0.0875 | 0.0875 | 0.2000 |  | 0.4 | ML |
| 129 | H | L | M | L | M |  | 0.1250 | 0.0000 | 0.0875 | 0.0000 | 0.2000 |  | 0.4 | ML |
| 131 | M | M | M | L | M |  | 0.0625 | 0.0625 | 0.0875 | 0.0000 | 0.2000 |  | 0.4 | ML |
| 133 | L | H | M | L | M |  | 0.0000 | 0.1250 | 0.0875 | 0.0000 | 0.2000 |  | 0.4 | ML |
| 141 | M | L | L | H | M |  | 0.0625 | 0.0000 | 0.0000 | 0.1750 | 0.2000 |  | 0.4 | ML |
| 143 | L | M | L | H | M |  | 0.0000 | 0.0625 | 0.0000 | 0.1750 | 0.2000 |  | 0.4 | ML |
| 144 | L | L | L | H | M |  | 0.0000 | 0.0000 | 0.0000 | 0.1750 | 0.2000 |  | 0.4 | ML |
| 147 | H | L | L | M | M |  | 0.1250 | 0.0000 | 0.0000 | 0.0875 | 0.2000 |  | 0.4 | ML |
| 149 | M | M | L | M | M |  | 0.0625 | 0.0625 | 0.0000 | 0.0875 | 0.2000 |  | 0.4 | ML |
| 151 | L | H | L | M | M |  | 0.0000 | 0.1250 | 0.0000 | 0.0875 | 0.2000 |  | 0.4 | ML |
| 154 | H | H | L | L | M |  | 0.1250 | 0.1250 | 0.0000 | 0.0000 | 0.2000 |  | 0.5 | M |
| 155 | H | M | L | L | M |  | 0.1250 | 0.0625 | 0.0000 | 0.0000 | 0.2000 |  | 0.4 | ML |
| 157 | M | H | L | L | M |  | 0.0625 | 0.1250 | 0.0000 | 0.0000 | 0.2000 |  | 0.4 | ML |
| 168 | M | L | H | H | L |  | 0.0625 | 0.0000 | 0.1750 | 0.1750 | 0.0000 |  | 0.4 | ML |
| 170 | L | M | H | H | L |  | 0.0000 | 0.0625 | 0.1750 | 0.1750 | 0.0000 |  | 0.4 | ML |
| 173 | H | M | H | M | L |  | 0.1250 | 0.0625 | 0.1750 | 0.0875 | 0.0000 |  | 0.5 | M |
| 174 | H | L | H | M | L |  | 0.1250 | 0.0000 | 0.1750 | 0.0875 | 0.0000 |  | 0.4 | ML |
| 175 | M | H | H | M | L |  | 0.0625 | 0.1250 | 0.1750 | 0.0875 | 0.0000 |  | 0.5 | M |
| 176 | M | M | H | M | L |  | 0.0625 | 0.0625 | 0.1750 | 0.0875 | 0.0000 |  | 0.4 | ML |
| 178 | L | H | H | M | L |  | 0.0000 | 0.1250 | 0.1750 | 0.0875 | 0.0000 |  | 0.4 | ML |
| 181 | H | H | H | L | L |  | 0.1250 | 0.1250 | 0.1750 | 0.0000 | 0.0000 |  | 0.4 | ML |
| 182 | H | M | H | L | L |  | 0.1250 | 0.0625 | 0.1750 | 0.0000 | 0.0000 |  | 0.4 | ML |
| 184 | M | H | H | L | L |  | 0.0625 | 0.1250 | 0.1750 | 0.0000 | 0.0000 |  | 0.4 | ML |
| 191 | H | M | M | H | L |  | 0.1250 | 0.0625 | 0.0875 | 0.1750 | 0.0000 |  | 0.5 | M |
| 192 | H | L | M | H | L |  | 0.1250 | 0.0000 | 0.0875 | 0.1750 | 0.0000 |  | 0.4 | ML |
| 193 | M | H | M | H | L |  | 0.0625 | 0.1250 | 0.0875 | 0.1750 | 0.0000 |  | 0.5 | M |
| 194 | M | M | M | H | L |  | 0.0625 | 0.0625 | 0.0875 | 0.1750 | 0.0000 |  | 0.4 | ML |
| 196 | L | H | M | H | L |  | 0.0000 | 0.1250 | 0.0875 | 0.1750 | 0.0000 |  | 0.4 | ML |
| 199 | H | H | M | M | L |  | 0.1250 | 0.1250 | 0.0875 | 0.0875 | 0.0000 |  | 0.4 | ML |
| 200 | H | M | M | M | L |  | 0.1250 | 0.0625 | 0.0875 | 0.0875 | 0.0000 |  | 0.4 | ML |
| 202 | M | H | M | M | L |  | 0.0625 | 0.1250 | 0.0875 | 0.0875 | 0.0000 |  | 0.4 | ML |
| 217 | H | H | L | H | L |  | 0.1250 | 0.1250 | 0.0000 | 0.1750 | 0.0000 |  | 0.4 | ML |
| 218 | H | M | L | H | L |  | 0.1250 | 0.0625 | 0.0000 | 0.1750 | 0.0000 |  | 0.4 | ML |
| 220 | M | H | L | H | L |  | 0.0625 | 0.1250 | 0.0000 | 0.1750 | 0.0000 |  | 0.4 | ML |
| 132 | M | L | M | L | M |  | 0.0625 | 0.0000 | 0.0875 | 0.0000 | 0.2000 |  | 0.4 | ML |
| 134 | L | M | M | L | M |  | 0.0000 | 0.0625 | 0.0875 | 0.0000 | 0.2000 |  | 0.4 | ML |
| 135 | L | L | M | L | M |  | 0.0000 | 0.0000 | 0.0875 | 0.0000 | 0.2000 |  | 0.3 | L |
| 150 | M | L | L | M | M |  | 0.0625 | 0.0000 | 0.0000 | 0.0875 | 0.2000 |  | 0.4 | ML |
| 152 | L | M | L | M | M |  | 0.0000 | 0.0625 | 0.0000 | 0.0875 | 0.2000 |  | 0.4 | ML |
| 153 | L | L | L | M | M |  | 0.0000 | 0.0000 | 0.0000 | 0.0875 | 0.2000 |  | 0.3 | L |
| 156 | H | L | L | L | M |  | 0.1250 | 0.0000 | 0.0000 | 0.0000 | 0.2000 |  | 0.3 | L |
| 158 | M | M | L | L | M |  | 0.0625 | 0.0625 | 0.0000 | 0.0000 | 0.2000 |  | 0.3 | L |
| 159 | M | L | L | L | M |  | 0.0625 | 0.0000 | 0.0000 | 0.0000 | 0.2000 |  | 0.3 | L |
| 160 | L | H | L | L | M |  | 0.0000 | 0.1250 | 0.0000 | 0.0000 | 0.2000 |  | 0.3 | L |
| 161 | L | M | L | L | M |  | 0.0000 | 0.0625 | 0.0000 | 0.0000 | 0.2000 |  | 0.3 | L |
| 171 | L | L | H | H | L |  | 0.0000 | 0.0000 | 0.1750 | 0.1750 | 0.0000 |  | 0.4 | ML |
| 177 | M | L | H | M | L |  | 0.0625 | 0.0000 | 0.1750 | 0.0875 | 0.0000 |  | 0.3 | L |
| 179 | L | M | H | M | L |  | 0.0000 | 0.0625 | 0.1750 | 0.0875 | 0.0000 |  | 0.3 | L |
| 180 | L | L | H | M | L |  | 0.0000 | 0.0000 | 0.1750 | 0.0875 | 0.0000 |  | 0.3 | L |
| 183 | H | L | H | L | L |  | 0.1250 | 0.0000 | 0.1750 | 0.0000 | 0.0000 |  | 0.3 | L |
| 185 | M | M | H | L | L |  | 0.0625 | 0.0625 | 0.1750 | 0.0000 | 0.0000 |  | 0.3 | L |
| 187 | L | H | H | L | L |  | 0.0000 | 0.1250 | 0.1750 | 0.0000 | 0.0000 |  | 0.3 | L |
| 195 | M | L | M | H | L |  | 0.0625 | 0.0000 | 0.0875 | 0.1750 | 0.0000 |  | 0.3 | L |
| 197 | L | M | M | H | L |  | 0.0000 | 0.0625 | 0.0875 | 0.1750 | 0.0000 |  | 0.3 | L |
| 198 | L | L | M | H | L |  | 0.0000 | 0.0000 | 0.0875 | 0.1750 | 0.0000 |  | 0.3 | L |
| 201 | H | L | M | M | L |  | 0.1250 | 0.0000 | 0.0875 | 0.0875 | 0.0000 |  | 0.3 | L |
| 203 | M | M | M | M | L |  | 0.0625 | 0.0625 | 0.0875 | 0.0875 | 0.0000 |  | 0.3 | L |
| 205 | L | H | M | M | L |  | 0.0000 | 0.1250 | 0.0875 | 0.0875 | 0.0000 |  | 0.3 | L |
| 208 | H | H | M | L | L |  | 0.1250 | 0.1250 | 0.0875 | 0.0000 | 0.0000 |  | 0.3 | L |
| 209 | H | M | M | L | L |  | 0.1250 | 0.0625 | 0.0875 | 0.0000 | 0.0000 |  | 0.3 | L |
| 211 | M | H | M | L | L |  | 0.0625 | 0.1250 | 0.0875 | 0.0000 | 0.0000 |  | 0.3 | L |
| 219 | H | L | L | H | L |  | 0.1250 | 0.0000 | 0.0000 | 0.1750 | 0.0000 |  | 0.3 | L |
| 221 | M | M | L | H | L |  | 0.0625 | 0.0625 | 0.0000 | 0.1750 | 0.0000 |  | 0.3 | L |
| 223 | L | H | L | H | L |  | 0.0000 | 0.1250 | 0.0000 | 0.1750 | 0.0000 |  | 0.3 | L |
| 226 | H | H | L | M | L |  | 0.1250 | 0.1250 | 0.0000 | 0.0875 | 0.0000 |  | 0.3 | L |
| 227 | H | M | L | M | L |  | 0.1250 | 0.0625 | 0.0000 | 0.0875 | 0.0000 |  | 0.3 | L |
| 229 | M | H | L | M | L |  | 0.0625 | 0.1250 | 0.0000 | 0.0875 | 0.0000 |  | 0.3 | L |
| 162 | L | L | L | L | M |  | 0.0000 | 0.0000 | 0.0000 | 0.0000 | 0.2000 |  | 0.2 | VL |
| 186 | M | L | H | L | L |  | 0.0625 | 0.0000 | 0.1750 | 0.0000 | 0.0000 |  | 0.2 | VL |
| 188 | L | M | H | L | L |  | 0.0000 | 0.0625 | 0.1750 | 0.0000 | 0.0000 |  | 0.2 | VL |
| 189 | L | L | H | L | L |  | 0.0000 | 0.0000 | 0.1750 | 0.0000 | 0.0000 |  | 0.2 | VL |
| 204 | M | L | M | M | L |  | 0.0625 | 0.0000 | 0.0875 | 0.0875 | 0.0000 |  | 0.2 | VL |
| 206 | L | M | M | M | L |  | 0.0000 | 0.0625 | 0.0875 | 0.0875 | 0.0000 |  | 0.2 | VL |
| 207 | L | L | M | M | L |  | 0.0000 | 0.0000 | 0.0875 | 0.0875 | 0.0000 |  | 0.2 | VL |
| 210 | H | L | M | L | L |  | 0.1250 | 0.0000 | 0.0875 | 0.0000 | 0.0000 |  | 0.2 | VL |
| 212 | M | M | M | L | L |  | 0.0625 | 0.0625 | 0.0875 | 0.0000 | 0.0000 |  | 0.2 | VL |
| 214 | L | H | M | L | L |  | 0.0000 | 0.1250 | 0.0875 | 0.0000 | 0.0000 |  | 0.2 | VL |
| 222 | M | L | L | H | L |  | 0.0625 | 0.0000 | 0.0000 | 0.1750 | 0.0000 |  | 0.2 | VL |
| 224 | L | M | L | H | L |  | 0.0000 | 0.0625 | 0.0000 | 0.1750 | 0.0000 |  | 0.2 | VL |
| 225 | L | L | L | H | L |  | 0.0000 | 0.0000 | 0.0000 | 0.1750 | 0.0000 |  | 0.2 | VL |
| 228 | H | L | L | M | L |  | 0.1250 | 0.0000 | 0.0000 | 0.0875 | 0.0000 |  | 0.2 | VL |
| 230 | M | M | L | M | L |  | 0.0625 | 0.0625 | 0.0000 | 0.0875 | 0.0000 |  | 0.2 | VL |
| 232 | L | H | L | M | L |  | 0.0000 | 0.1250 | 0.0000 | 0.0875 | 0.0000 |  | 0.2 | VL |
| 235 | H | H | L | L | L |  | 0.1250 | 0.1250 | 0.0000 | 0.0000 | 0.0000 |  | 0.3 | L |
| 236 | H | M | L | L | L |  | 0.1250 | 0.0625 | 0.0000 | 0.0000 | 0.0000 |  | 0.2 | VL |
| 238 | M | H | L | L | L |  | 0.0625 | 0.1250 | 0.0000 | 0.0000 | 0.0000 |  | 0.2 | VL |
| 213 | M | L | M | L | L |  | 0.0625 | 0.0000 | 0.0875 | 0.0000 | 0.0000 |  | 0.2 | VL |
| 215 | L | M | M | L | L |  | 0.0000 | 0.0625 | 0.0875 | 0.0000 | 0.0000 |  | 0.2 | VL |
| 216 | L | L | M | L | L |  | 0.0000 | 0.0000 | 0.0875 | 0.0000 | 0.0000 |  | 0.1 | VVL |
| 231 | M | L | L | M | L |  | 0.0625 | 0.0000 | 0.0000 | 0.0875 | 0.0000 |  | 0.2 | VL |
| 233 | L | M | L | M | L |  | 0.0000 | 0.0625 | 0.0000 | 0.0875 | 0.0000 |  | 0.2 | VL |
| 234 | L | L | L | M | L |  | 0.0000 | 0.0000 | 0.0000 | 0.0875 | 0.0000 |  | 0.1 | VVL |
| 237 | H | L | L | L | L |  | 0.1250 | 0.0000 | 0.0000 | 0.0000 | 0.0000 |  | 0.1 | VVL |
| 239 | M | M | L | L | L |  | 0.0625 | 0.0625 | 0.0000 | 0.0000 | 0.0000 |  | 0.1 | VVL |
| 240 | M | L | L | L | L |  | 0.0625 | 0.0000 | 0.0000 | 0.0000 | 0.0000 |  | 0.1 | VVL |
| 241 | L | H | L | L | L |  | 0.0000 | 0.1250 | 0.0000 | 0.0000 | 0.0000 |  | 0.1 | VVL |
| 242 | L | M | L | L | L |  | 0.0000 | 0.0625 | 0.0000 | 0.0000 | 0.0000 |  | 0.1 | VVL |
| 243 | L | L | L | L | L |  | 0.0000 | 0.0000 | 0.0000 | 0.0000 | 0.0000 |  | 0.0 | VVVL |

**Table S5** The grades and rank by adopting different approaches

| Experiment | Grey relational analysis | | Triangular + gaussian | | Triangular | |
| --- | --- | --- | --- | --- | --- | --- |
| No. | GRG | Order | GFG | Order | GFG | Order |
| 1 | 0.727 | 8 | 0.772 | 7 | 0.82 | 5 |
| 2 | 0.903 | 1 | 0.938 | 1 | 0.7 | 10 |
| 3 | 0.801 | 4 | 0.86 | 4 | 0.88 | 2 |
| 4 | 0.740 | 6 | 0.785 | 6 | 0.764 | 6 |
| 5 | 0.710 | 9 | 0.736 | 10 | 0.7 | 10 |
| 6 | 0.857 | 2 | 0.88 | 3 | 0.88 | 2 |
| 7 | 0.629 | 15 | 0.677 | 13 | 0.653 | 15 |
| 8 | 0.560 | 21 | 0.584 | 21 | 0.568 | 23 |
| 9 | 0.603 | 18 | 0.641 | 16 | 0.64 | 17 |
| 10 | 0.639 | 14 | 0.661 | 15 | 0.651 | 16 |
| 11 | 0.595 | 19 | 0.62 | 19 | 0.615 | 20 |
| 12 | 0.660 | 12 | 0.673 | 14 | 0.7 | 10 |
| 13 | 0.495 | 24 | 0.516 | 25 | 0.516 | 25 |
| 14 | 0.557 | 22 | 0.578 | 23 | 0.578 | 22 |
| 15 | 0.467 | 25 | 0.521 | 24 | 0.52 | 24 |
| 16 | 0.737 | 7 | 0.755 | 9 | 0.76 | 7 |
| 17 | 0.561 | 20 | 0.636 | 18 | 0.64 | 17 |
| 18 | 0.604 | 17 | 0.64 | 17 | 0.64 | 17 |
| 19 | 0.696 | 11 | 0.721 | 11 | 0.7 | 10 |
| 20 | 0.644 | 13 | 0.71 | 12 | 0.716 | 9 |
| 21 | 0.620 | 16 | 0.616 | 20 | 0.7 | 10 |
| 22 | 0.763 | 5 | 0.805 | 5 | 0.833 | 4 |
| 23 | 0.519 | 23 | 0.582 | 22 | 0.58 | 21 |
| 24 | 0.698 | 10 | 0.759 | 8 | 0.76 | 7 |
| 25 | 0.844 | 3 | 0.892 | 2 | 0.943 | 1 |

**Table S6** The program of “Triangular + gaussian” and “Triangular”

| Triangular + gaussian | Triangular |
| --- | --- |
| [System]  Name='Triangular + gaussian'  Type='mamdani'  Version=2.0  NumInputs=5  NumOutputs=1  NumRules=243  AndMethod='min'  OrMethod='max'  ImpMethod='min'  AggMethod='max'  DefuzzMethod='centroid'  [Input1]  Name='RSS'  Range=[0.3 1]  NumMFs=3  MF1='Small':'gaussmf',[0.07432 0.475]  MF2='Mid':'gaussmf',[0.07432 0.65]  MF3='Big':'gaussmf',[0.07432 0.825]  [Input2]  Name='RSC'  Range=[0.3 1]  NumMFs=3  MF1='Small':'gaussmf',[0.07432 0.475]  MF2='Mid':'gaussmf',[0.07432 0.65]  MF3='Big':'gaussmf',[0.07432 0.825]  [Input3]  Name='WS'  Range=[0.3 1]  NumMFs=3  MF1='Small':'gaussmf',[0.07432 0.475]  MF2='Mid':'gaussmf',[0.07432 0.65]  MF3='Big':'gaussmf',[0.07432 0.825]  [Input4]  Name='WC'  Range=[0.3 1]  NumMFs=3  MF1='Small':'gaussmf',[0.07432 0.475]  MF2='Mid':'gaussmf',[0.07432 0.65]  MF3='Big':'gaussmf',[0.07432 0.825]  [Input5]  Name='RFM'  Range=[0.3 1]  NumMFs=3  MF1='Small':'gaussmf',[0.07432 0.475]  MF2='Mid':'gaussmf',[0.07432 0.65]  MF3='Big':'gaussmf',[0.07432 0.825]  [Output1]  Name='GFG'  Range=[0.4 1]  NumMFs=11  MF1='VL':'trimf',[0.46 0.52 0.58]  MF2='M':'trimf',[0.64 0.7 0.76]  MF3='VVH':'trimf',[0.88 0.94 1]  MF4='VVL':'trimf',[0.4 0.46 0.52]  MF5='L':'trimf',[0.52 0.58 0.64]  MF6='VH':'trimf',[0.82 0.88 0.94]  MF7='ML':'trimf',[0.58 0.64 0.7]  MF8='MH':'trimf',[0.7 0.76 0.82]  MF9='H':'trimf',[0.76 0.82 0.88]  MF10='VVVL':'trimf',[0.34 0.4 0.46]  MF11='VVVH':'trimf',[0.94 1 1.06]  [Rules]  3 3 3 3 3, 11 (1) : 1  3 2 3 3 3, 3 (1) : 1  3 1 3 3 3, 3 (1) : 1  2 3 3 3 3, 3 (1) : 1  2 2 3 3 3, 3 (1) : 1  1 3 3 3 3, 3 (1) : 1  3 3 3 2 3, 3 (1) : 1  3 3 2 3 3, 3 (1) : 1  2 1 3 3 3, 6 (1) : 1  1 2 3 3 3, 6 (1) : 1  3 2 3 2 3, 6 (1) : 1  3 1 3 2 3, 6 (1) : 1  2 3 3 2 3, 6 (1) : 1  2 2 3 2 3, 6 (1) : 1  1 3 3 2 3, 6 (1) : 1  3 3 3 1 3, 6 (1) : 1  3 2 3 1 3, 6 (1) : 1  2 3 3 1 3, 6 (1) : 1  3 2 2 3 3, 6 (1) : 1  3 1 2 3 3, 6 (1) : 1  2 3 2 3 3, 6 (1) : 1  2 2 2 3 3, 6 (1) : 1  1 3 2 3 3, 6 (1) : 1  3 3 2 2 3, 6 (1) : 1  3 2 2 2 3, 6 (1) : 1  2 3 2 2 3, 6 (1) : 1  3 3 1 3 3, 6 (1) : 1  3 2 1 3 3, 6 (1) : 1  2 3 1 3 3, 6 (1) : 1  3 3 3 3 2, 6 (1) : 1  1 1 3 3 3, 9 (1) : 1  2 1 3 2 3, 9 (1) : 1  1 2 3 2 3, 9 (1) : 1  1 1 3 2 3, 9 (1) : 1  3 1 3 1 3, 9 (1) : 1  2 2 3 1 3, 9 (1) : 1  1 3 3 1 3, 9 (1) : 1  2 1 2 3 3, 9 (1) : 1  1 2 2 3 3, 9 (1) : 1  1 1 2 3 3, 9 (1) : 1  3 1 2 2 3, 9 (1) : 1  2 2 2 2 3, 9 (1) : 1  1 3 2 2 3, 9 (1) : 1  3 3 2 1 3, 9 (1) : 1  3 2 2 1 3, 9 (1) : 1  2 3 2 1 3, 9 (1) : 1  3 1 1 3 3, 9 (1) : 1  2 2 1 3 3, 9 (1) : 1  1 3 1 3 3, 9 (1) : 1  3 3 1 2 3, 9 (1) : 1  3 2 1 2 3, 9 (1) : 1  2 3 1 2 3, 9 (1) : 1  3 2 3 3 2, 9 (1) : 1  3 1 3 3 2, 9 (1) : 1  2 3 3 3 2, 9 (1) : 1  2 2 3 3 2, 9 (1) : 1  1 3 3 3 2, 9 (1) : 1  3 3 3 2 2, 9 (1) : 1  3 3 2 3 2, 9 (1) : 1  2 1 3 1 3, 8 (1) : 1  1 2 3 1 3, 8 (1) : 1  1 1 3 1 3, 8 (1) : 1  2 1 2 2 3, 8 (1) : 1  1 2 2 2 3, 8 (1) : 1  1 1 2 2 3, 8 (1) : 1  3 1 2 1 3, 8 (1) : 1  2 2 2 1 3, 8 (1) : 1  1 3 2 1 3, 8 (1) : 1  2 1 1 3 3, 8 (1) : 1  1 2 1 3 3, 8 (1) : 1  1 1 1 3 3, 8 (1) : 1  3 1 1 2 3, 8 (1) : 1  2 2 1 2 3, 8 (1) : 1  1 3 1 2 3, 8 (1) : 1  3 3 1 1 3, 8 (1) : 1  3 2 1 1 3, 8 (1) : 1  2 3 1 1 3, 8 (1) : 1  2 1 3 3 2, 8 (1) : 1  1 2 3 3 2, 8 (1) : 1  3 2 3 2 2, 8 (1) : 1  3 1 3 2 2, 8 (1) : 1  2 3 3 2 2, 8 (1) : 1  2 2 3 2 2, 8 (1) : 1  1 3 3 2 2, 8 (1) : 1  3 3 3 1 2, 8 (1) : 1  3 2 3 1 2, 8 (1) : 1  2 3 3 1 2, 8 (1) : 1  3 2 2 3 2, 8 (1) : 1  3 1 2 3 2, 8 (1) : 1  2 3 2 3 2, 8 (1) : 1  2 2 2 3 2, 8 (1) : 1  1 3 2 3 2, 8 (1) : 1  3 3 2 2 2, 8 (1) : 1  3 2 2 2 2, 8 (1) : 1  2 3 2 2 2, 8 (1) : 1  3 3 1 3 2, 8 (1) : 1  3 2 1 3 2, 8 (1) : 1  2 3 1 3 2, 8 (1) : 1  3 3 3 3 1, 8 (1) : 1  2 1 2 1 3, 2 (1) : 1  1 2 2 1 3, 2 (1) : 1  1 1 2 1 3, 2 (1) : 1  2 1 1 2 3, 2 (1) : 1  1 2 1 2 3, 2 (1) : 1  1 1 1 2 3, 2 (1) : 1  3 1 1 1 3, 2 (1) : 1  2 2 1 1 3, 2 (1) : 1  2 1 1 1 3, 2 (1) : 1  1 3 1 1 3, 2 (1) : 1  1 2 1 1 3, 2 (1) : 1  1 1 3 3 2, 2 (1) : 1  2 1 3 2 2, 2 (1) : 1  1 2 3 2 2, 2 (1) : 1  1 1 3 2 2, 2 (1) : 1  3 1 3 1 2, 2 (1) : 1  2 2 3 1 2, 2 (1) : 1  1 3 3 1 2, 2 (1) : 1  2 1 2 3 2, 2 (1) : 1  1 2 2 3 2, 2 (1) : 1  1 1 2 3 2, 2 (1) : 1  3 1 2 2 2, 2 (1) : 1  2 2 2 2 2, 2 (1) : 1  1 3 2 2 2, 2 (1) : 1  3 3 2 1 2, 2 (1) : 1  3 2 2 1 2, 2 (1) : 1  2 3 2 1 2, 2 (1) : 1  3 1 1 3 2, 2 (1) : 1  2 2 1 3 2, 2 (1) : 1  1 3 1 3 2, 2 (1) : 1  3 3 1 2 2, 2 (1) : 1  3 2 1 2 2, 2 (1) : 1  2 3 1 2 2, 2 (1) : 1  3 2 3 3 1, 2 (1) : 1  3 1 3 3 1, 2 (1) : 1  2 3 3 3 1, 2 (1) : 1  2 2 3 3 1, 2 (1) : 1  1 3 3 3 1, 2 (1) : 1  3 3 3 2 1, 2 (1) : 1  3 3 2 3 1, 2 (1) : 1  1 1 1 1 3, 7 (1) : 1  2 1 3 1 2, 7 (1) : 1  1 2 3 1 2, 7 (1) : 1  1 1 3 1 2, 7 (1) : 1  2 1 2 2 2, 7 (1) : 1  1 2 2 2 2, 7 (1) : 1  1 1 2 2 2, 7 (1) : 1  3 1 2 1 2, 7 (1) : 1  2 2 2 1 2, 7 (1) : 1  1 3 2 1 2, 7 (1) : 1  2 1 1 3 2, 7 (1) : 1  1 2 1 3 2, 7 (1) : 1  1 1 1 3 2, 7 (1) : 1  3 1 1 2 2, 7 (1) : 1  2 2 1 2 2, 7 (1) : 1  1 3 1 2 2, 7 (1) : 1  3 3 1 1 2, 7 (1) : 1  3 2 1 1 2, 7 (1) : 1  2 3 1 1 2, 7 (1) : 1  2 1 3 3 1, 7 (1) : 1  1 2 3 3 1, 7 (1) : 1  3 2 3 2 1, 7 (1) : 1  3 1 3 2 1, 7 (1) : 1  2 3 3 2 1, 7 (1) : 1  2 2 3 2 1, 7 (1) : 1  1 3 3 2 1, 7 (1) : 1  3 3 3 1 1, 7 (1) : 1  3 2 3 1 1, 7 (1) : 1  2 3 3 1 1, 7 (1) : 1  3 2 2 3 1, 7 (1) : 1  3 1 2 3 1, 7 (1) : 1  2 3 2 3 1, 7 (1) : 1  2 2 2 3 1, 7 (1) : 1  1 3 2 3 1, 7 (1) : 1  3 3 2 2 1, 7 (1) : 1  3 2 2 2 1, 7 (1) : 1  2 3 2 2 1, 7 (1) : 1  3 3 1 3 1, 7 (1) : 1  3 2 1 3 1, 7 (1) : 1  2 3 1 3 1, 7 (1) : 1  2 1 2 1 2, 5 (1) : 1  1 2 2 1 2, 5 (1) : 1  1 1 2 1 2, 5 (1) : 1  2 1 1 2 2, 5 (1) : 1  1 2 1 2 2, 5 (1) : 1  1 1 1 2 2, 5 (1) : 1  3 1 1 1 2, 5 (1) : 1  2 2 1 1 2, 5 (1) : 1  2 1 1 1 2, 5 (1) : 1  1 3 1 1 2, 5 (1) : 1  1 2 1 1 2, 5 (1) : 1  1 1 3 3 1, 5 (1) : 1  2 1 3 2 1, 5 (1) : 1  1 2 3 2 1, 5 (1) : 1  1 1 3 2 1, 5 (1) : 1  3 1 3 1 1, 5 (1) : 1  2 2 3 1 1, 5 (1) : 1  1 3 3 1 1, 5 (1) : 1  2 1 2 3 1, 5 (1) : 1  1 2 2 3 1, 5 (1) : 1  1 1 2 3 1, 5 (1) : 1  3 1 2 2 1, 5 (1) : 1  2 2 2 2 1, 5 (1) : 1  1 3 2 2 1, 5 (1) : 1  3 3 2 1 1, 5 (1) : 1  3 2 2 1 1, 5 (1) : 1  2 3 2 1 1, 5 (1) : 1  3 1 1 3 1, 5 (1) : 1  2 2 1 3 1, 5 (1) : 1  1 3 1 3 1, 5 (1) : 1  3 3 1 2 1, 5 (1) : 1  3 2 1 2 1, 5 (1) : 1  2 3 1 2 1, 5 (1) : 1  1 1 1 1 2, 1 (1) : 1  2 1 3 1 1, 1 (1) : 1  1 2 3 1 1, 1 (1) : 1  1 1 3 1 1, 1 (1) : 1  2 1 2 2 1, 1 (1) : 1  1 2 2 2 1, 1 (1) : 1  1 1 2 2 1, 1 (1) : 1  3 1 2 1 1, 1 (1) : 1  2 2 2 1 1, 1 (1) : 1  1 3 2 1 1, 1 (1) : 1  2 1 1 3 1, 1 (1) : 1  1 2 1 3 1, 1 (1) : 1  1 1 1 3 1, 1 (1) : 1  3 1 1 2 1, 1 (1) : 1  2 2 1 2 1, 1 (1) : 1  1 3 1 2 1, 1 (1) : 1  3 3 1 1 1, 1 (1) : 1  3 2 1 1 1, 1 (1) : 1  2 3 1 1 1, 1 (1) : 1  2 1 2 1 1, 4 (1) : 1  1 2 2 1 1, 4 (1) : 1  1 1 2 1 1, 4 (1) : 1  2 1 1 2 1, 4 (1) : 1  1 2 1 2 1, 4 (1) : 1  1 1 1 2 1, 4 (1) : 1  3 1 1 1 1, 4 (1) : 1  2 2 1 1 1, 4 (1) : 1  2 1 1 1 1, 4 (1) : 1  1 3 1 1 1, 4 (1) : 1  1 2 1 1 1, 4 (1) : 1  1 1 1 1 1, 10 (1) : 1 | [System]  Name='Triangular'  Type='mamdani'  Version=2.0  NumInputs=5  NumOutputs=1  NumRules=243  AndMethod='min'  OrMethod='max'  ImpMethod='min'  AggMethod='max'  DefuzzMethod='centroid'  [Input1]  Name='RSS'  Range=[0.3 1]  NumMFs=3  MF1='Small':'trimf',[0.3 0.475 0.65]  MF2='Mid':'trimf',[0.475 0.65 0.825]  MF3='Big':'trimf',[0.65 0.825 1]  [Input2]  Name='RSC'  Range=[0.3 1]  NumMFs=3  MF1='Small':'trimf',[0.3 0.475 0.65]  MF2='Mid':'trimf',[0.475 0.65 0.825]  MF3='Big':'trimf',[0.65 0.825 1]  [Input3]  Name='WS'  Range=[0.3 1]  NumMFs=3  MF1='Small':'trimf',[0.3 0.475 0.65]  MF2='Mid':'trimf',[0.475 0.65 0.825]  MF3='Big':'trimf',[0.65 0.825 1]  [Input4]  Name='WC'  Range=[0.3 1]  NumMFs=3  MF1='Small':'trimf',[0.3 0.475 0.65]  MF2='Mid':'trimf',[0.475 0.65 0.825]  MF3='Big':'trimf',[0.65 0.825 1]  [Input5]  Name='RFM'  Range=[0.3 1]  NumMFs=3  MF1='Small':'trimf',[0.3 0.475 0.65]  MF2='Mid':'trimf',[0.475 0.65 0.825]  MF3='Big':'trimf',[0.65 0.825 1]  [Output1]  Name='GFG'  Range=[0.4 1]  NumMFs=11  MF1='VL':'trimf',[0.46 0.52 0.58]  MF2='M':'trimf',[0.64 0.7 0.76]  MF3='VVH':'trimf',[0.88 0.94 1]  MF4='VVL':'trimf',[0.4 0.46 0.52]  MF5='L':'trimf',[0.52 0.58 0.64]  MF6='VH':'trimf',[0.82 0.88 0.94]  MF7='ML':'trimf',[0.58 0.64 0.7]  MF8='MH':'trimf',[0.7 0.76 0.82]  MF9='H':'trimf',[0.76 0.82 0.88]  MF10='VVVL':'trimf',[0.34 0.4 0.46]  MF11='VVVH':'trimf',[0.94 1 1.06]  [Rules]  3 3 3 3 3, 11 (1) : 1  3 2 3 3 3, 3 (1) : 1  3 1 3 3 3, 3 (1) : 1  2 3 3 3 3, 3 (1) : 1  2 2 3 3 3, 3 (1) : 1  1 3 3 3 3, 3 (1) : 1  3 3 3 2 3, 3 (1) : 1  3 3 2 3 3, 3 (1) : 1  2 1 3 3 3, 6 (1) : 1  1 2 3 3 3, 6 (1) : 1  3 2 3 2 3, 6 (1) : 1  3 1 3 2 3, 6 (1) : 1  2 3 3 2 3, 6 (1) : 1  2 2 3 2 3, 6 (1) : 1  1 3 3 2 3, 6 (1) : 1  3 3 3 1 3, 6 (1) : 1  3 2 3 1 3, 6 (1) : 1  2 3 3 1 3, 6 (1) : 1  3 2 2 3 3, 6 (1) : 1  3 1 2 3 3, 6 (1) : 1  2 3 2 3 3, 6 (1) : 1  2 2 2 3 3, 6 (1) : 1  1 3 2 3 3, 6 (1) : 1  3 3 2 2 3, 6 (1) : 1  3 2 2 2 3, 6 (1) : 1  2 3 2 2 3, 6 (1) : 1  3 3 1 3 3, 6 (1) : 1  3 2 1 3 3, 6 (1) : 1  2 3 1 3 3, 6 (1) : 1  3 3 3 3 2, 6 (1) : 1  1 1 3 3 3, 9 (1) : 1  2 1 3 2 3, 9 (1) : 1  1 2 3 2 3, 9 (1) : 1  1 1 3 2 3, 9 (1) : 1  3 1 3 1 3, 9 (1) : 1  2 2 3 1 3, 9 (1) : 1  1 3 3 1 3, 9 (1) : 1  2 1 2 3 3, 9 (1) : 1  1 2 2 3 3, 9 (1) : 1  1 1 2 3 3, 9 (1) : 1  3 1 2 2 3, 9 (1) : 1  2 2 2 2 3, 9 (1) : 1  1 3 2 2 3, 9 (1) : 1  3 3 2 1 3, 9 (1) : 1  3 2 2 1 3, 9 (1) : 1  2 3 2 1 3, 9 (1) : 1  3 1 1 3 3, 9 (1) : 1  2 2 1 3 3, 9 (1) : 1  1 3 1 3 3, 9 (1) : 1  3 3 1 2 3, 9 (1) : 1  3 2 1 2 3, 9 (1) : 1  2 3 1 2 3, 9 (1) : 1  3 2 3 3 2, 9 (1) : 1  3 1 3 3 2, 9 (1) : 1  2 3 3 3 2, 9 (1) : 1  2 2 3 3 2, 9 (1) : 1  1 3 3 3 2, 9 (1) : 1  3 3 3 2 2, 9 (1) : 1  3 3 2 3 2, 9 (1) : 1  2 1 3 1 3, 8 (1) : 1  1 2 3 1 3, 8 (1) : 1  1 1 3 1 3, 8 (1) : 1  2 1 2 2 3, 8 (1) : 1  1 2 2 2 3, 8 (1) : 1  1 1 2 2 3, 8 (1) : 1  3 1 2 1 3, 8 (1) : 1  2 2 2 1 3, 8 (1) : 1  1 3 2 1 3, 8 (1) : 1  2 1 1 3 3, 8 (1) : 1  1 2 1 3 3, 8 (1) : 1  1 1 1 3 3, 8 (1) : 1  3 1 1 2 3, 8 (1) : 1  2 2 1 2 3, 8 (1) : 1  1 3 1 2 3, 8 (1) : 1  3 3 1 1 3, 8 (1) : 1  3 2 1 1 3, 8 (1) : 1  2 3 1 1 3, 8 (1) : 1  2 1 3 3 2, 8 (1) : 1  1 2 3 3 2, 8 (1) : 1  3 2 3 2 2, 8 (1) : 1  3 1 3 2 2, 8 (1) : 1  2 3 3 2 2, 8 (1) : 1  2 2 3 2 2, 8 (1) : 1  1 3 3 2 2, 8 (1) : 1  3 3 3 1 2, 8 (1) : 1  3 2 3 1 2, 8 (1) : 1  2 3 3 1 2, 8 (1) : 1  3 2 2 3 2, 8 (1) : 1  3 1 2 3 2, 8 (1) : 1  2 3 2 3 2, 8 (1) : 1  2 2 2 3 2, 8 (1) : 1  1 3 2 3 2, 8 (1) : 1  3 3 2 2 2, 8 (1) : 1  3 2 2 2 2, 8 (1) : 1  2 3 2 2 2, 8 (1) : 1  3 3 1 3 2, 8 (1) : 1  3 2 1 3 2, 8 (1) : 1  2 3 1 3 2, 8 (1) : 1  3 3 3 3 1, 8 (1) : 1  2 1 2 1 3, 2 (1) : 1  1 2 2 1 3, 2 (1) : 1  1 1 2 1 3, 2 (1) : 1  2 1 1 2 3, 2 (1) : 1  1 2 1 2 3, 2 (1) : 1  1 1 1 2 3, 2 (1) : 1  3 1 1 1 3, 2 (1) : 1  2 2 1 1 3, 2 (1) : 1  2 1 1 1 3, 2 (1) : 1  1 3 1 1 3, 2 (1) : 1  1 2 1 1 3, 2 (1) : 1  1 1 3 3 2, 2 (1) : 1  2 1 3 2 2, 2 (1) : 1  1 2 3 2 2, 2 (1) : 1  1 1 3 2 2, 2 (1) : 1  3 1 3 1 2, 2 (1) : 1  2 2 3 1 2, 2 (1) : 1  1 3 3 1 2, 2 (1) : 1  2 1 2 3 2, 2 (1) : 1  1 2 2 3 2, 2 (1) : 1  1 1 2 3 2, 2 (1) : 1  3 1 2 2 2, 2 (1) : 1  2 2 2 2 2, 2 (1) : 1  1 3 2 2 2, 2 (1) : 1  3 3 2 1 2, 2 (1) : 1  3 2 2 1 2, 2 (1) : 1  2 3 2 1 2, 2 (1) : 1  3 1 1 3 2, 2 (1) : 1  2 2 1 3 2, 2 (1) : 1  1 3 1 3 2, 2 (1) : 1  3 3 1 2 2, 2 (1) : 1  3 2 1 2 2, 2 (1) : 1  2 3 1 2 2, 2 (1) : 1  3 2 3 3 1, 2 (1) : 1  3 1 3 3 1, 2 (1) : 1  2 3 3 3 1, 2 (1) : 1  2 2 3 3 1, 2 (1) : 1  1 3 3 3 1, 2 (1) : 1  3 3 3 2 1, 2 (1) : 1  3 3 2 3 1, 2 (1) : 1  1 1 1 1 3, 7 (1) : 1  2 1 3 1 2, 7 (1) : 1  1 2 3 1 2, 7 (1) : 1  1 1 3 1 2, 7 (1) : 1  2 1 2 2 2, 7 (1) : 1  1 2 2 2 2, 7 (1) : 1  1 1 2 2 2, 7 (1) : 1  3 1 2 1 2, 7 (1) : 1  2 2 2 1 2, 7 (1) : 1  1 3 2 1 2, 7 (1) : 1  2 1 1 3 2, 7 (1) : 1  1 2 1 3 2, 7 (1) : 1  1 1 1 3 2, 7 (1) : 1  3 1 1 2 2, 7 (1) : 1  2 2 1 2 2, 7 (1) : 1  1 3 1 2 2, 7 (1) : 1  3 3 1 1 2, 7 (1) : 1  3 2 1 1 2, 7 (1) : 1  2 3 1 1 2, 7 (1) : 1  2 1 3 3 1, 7 (1) : 1  1 2 3 3 1, 7 (1) : 1  3 2 3 2 1, 7 (1) : 1  3 1 3 2 1, 7 (1) : 1  2 3 3 2 1, 7 (1) : 1  2 2 3 2 1, 7 (1) : 1  1 3 3 2 1, 7 (1) : 1  3 3 3 1 1, 7 (1) : 1  3 2 3 1 1, 7 (1) : 1  2 3 3 1 1, 7 (1) : 1  3 2 2 3 1, 7 (1) : 1  3 1 2 3 1, 7 (1) : 1  2 3 2 3 1, 7 (1) : 1  2 2 2 3 1, 7 (1) : 1  1 3 2 3 1, 7 (1) : 1  3 3 2 2 1, 7 (1) : 1  3 2 2 2 1, 7 (1) : 1  2 3 2 2 1, 7 (1) : 1  3 3 1 3 1, 7 (1) : 1  3 2 1 3 1, 7 (1) : 1  2 3 1 3 1, 7 (1) : 1  2 1 2 1 2, 5 (1) : 1  1 2 2 1 2, 5 (1) : 1  1 1 2 1 2, 5 (1) : 1  2 1 1 2 2, 5 (1) : 1  1 2 1 2 2, 5 (1) : 1  1 1 1 2 2, 5 (1) : 1  3 1 1 1 2, 5 (1) : 1  2 2 1 1 2, 5 (1) : 1  2 1 1 1 2, 5 (1) : 1  1 3 1 1 2, 5 (1) : 1  1 2 1 1 2, 5 (1) : 1  1 1 3 3 1, 5 (1) : 1  2 1 3 2 1, 5 (1) : 1  1 2 3 2 1, 5 (1) : 1  1 1 3 2 1, 5 (1) : 1  3 1 3 1 1, 5 (1) : 1  2 2 3 1 1, 5 (1) : 1  1 3 3 1 1, 5 (1) : 1  2 1 2 3 1, 5 (1) : 1  1 2 2 3 1, 5 (1) : 1  1 1 2 3 1, 5 (1) : 1  3 1 2 2 1, 5 (1) : 1  2 2 2 2 1, 5 (1) : 1  1 3 2 2 1, 5 (1) : 1  3 3 2 1 1, 5 (1) : 1  3 2 2 1 1, 5 (1) : 1  2 3 2 1 1, 5 (1) : 1  3 1 1 3 1, 5 (1) : 1  2 2 1 3 1, 5 (1) : 1  1 3 1 3 1, 5 (1) : 1  3 3 1 2 1, 5 (1) : 1  3 2 1 2 1, 5 (1) : 1  2 3 1 2 1, 5 (1) : 1  1 1 1 1 2, 1 (1) : 1  2 1 3 1 1, 1 (1) : 1  1 2 3 1 1, 1 (1) : 1  1 1 3 1 1, 1 (1) : 1  2 1 2 2 1, 1 (1) : 1  1 2 2 2 1, 1 (1) : 1  1 1 2 2 1, 1 (1) : 1  3 1 2 1 1, 1 (1) : 1  2 2 2 1 1, 1 (1) : 1  1 3 2 1 1, 1 (1) : 1  2 1 1 3 1, 1 (1) : 1  1 2 1 3 1, 1 (1) : 1  1 1 1 3 1, 1 (1) : 1  3 1 1 2 1, 1 (1) : 1  2 2 1 2 1, 1 (1) : 1  1 3 1 2 1, 1 (1) : 1  3 3 1 1 1, 1 (1) : 1  3 2 1 1 1, 1 (1) : 1  2 3 1 1 1, 1 (1) : 1  2 1 2 1 1, 4 (1) : 1  1 2 2 1 1, 4 (1) : 1  1 1 2 1 1, 4 (1) : 1  2 1 1 2 1, 4 (1) : 1  1 2 1 2 1, 4 (1) : 1  1 1 1 2 1, 4 (1) : 1  3 1 1 1 1, 4 (1) : 1  2 2 1 1 1, 4 (1) : 1  2 1 1 1 1, 4 (1) : 1  1 3 1 1 1, 4 (1) : 1  1 2 1 1 1, 4 (1) : 1  1 1 1 1 1, 10 (1) : 1 |
